# CD93 in macrophages: A novel target for atherosclerotic plaque imaging?

**DOI:** 10.1111/jcmm.17237

**Published:** 2022-02-15

**Authors:** Chen Su, Yeming Han, Bin Qu, Chao Zhang, Ting Liang, Feng Gao, Guihua Hou

**Affiliations:** ^1^ Key Laboratory for Experimental Teratology of the Ministry of Education and Research Center for Experimental Nuclear Medicine School of Basic Medical Sciences Cheeloo College of Medicine Shandong University Jinan China; ^2^ Radiology Department Qilu Hospital of Shandong University Jinan China

**Keywords:** atherosclerosis, CD93, macrophage, molecular imaging, radionuclide

## Abstract

Noninvasive imaging atherosclerotic (AS) plaque is of great importance for early diagnosis. Recently, CD93 in MΦ was linked to atherosclerosis development. Herein, we have investigated whether CD93 in MΦ is a potential novel target for atherosclerotic plaque imaging. CD93^hi^ and CD93^lo^ MΦ were prepared with or without LPS stimulation, before biological activity was evaluated. A rat AS model was produced with left carotid artery clamped. Whole‐body/ex vivo phosphor autoradiography of the artery and biodistribution were investigated after incorporation of ^3^H‐2‐DG into CD93^hi^ and CD93^lo^ MΦ or after ^125^I‐α‐CD93 (^125^I‐anti‐CD93mAb) injection. The plaque tissue was subjected to CD93/CD68 immunofluorescence/immunohistochemistry staining. CD93^hi^ and CD93^lo^ MΦ cells were successfully prepared without significant effect on bioactivity after incorporative labelled with ^3^H‐2‐DG. The AS model was successfully established. Biodistribution studies showed that adoptive transfer of ^3^H‐2‐DG‐CD93^hi^ MΦ or ^125^I‐ α‐CD93 injection resulted in accumulation of radioactivity within the atherosclerotic plaque in the clamped left carotid artery. T/NT (target/non‐target, left/right carotid artery) ratio was higher in the ^3^H‐2‐DG‐CD93^hi^ MΦ adoptive transfer group than in the ^3^H‐2‐DG‐CD93^lo^ MΦ group (*p* < .05). Plaque radioactivity in the ^125^I‐α‐CD93 injection group was significantly higher than in the ^125^I‐IgG control group (*p* < .01). The higher radioactivity accumulated in the clamped left carotid artery was confirmed by phosphor autoradiography. More importantly, CD93/CD68 double‐positive MΦ accumulated at the atherosclerotic plaque in ^3^H‐2‐DG‐CD93^hi^ MΦ adoptive transfer group, which correlated with plaque radioactivity (*r* = .99, *p* < .01). In summary, both adoptive‐transferred ^3^H‐2‐DG‐labelled CD93^hi^ MΦ and ^125^I‐α‐CD93 injection specifically targeted CD93 in atherosclerotic plaque. CD93 is a potential target in atherosclerotic plaque imaging.

## 1 INTRODUCTION

Atherosclerosis (AS) is a complex inflammatory disease in the arterial wall which can lead to myocardial infarction, stroke and peripheral vascular diseases.^1^ At present, diagnostic tools of advanced AS in the clinic include arteriography, Doppler ultrasound, computed tomography (CT) and coronary CT angiography (CCTA),^2^ which are effective for determining anatomical changes in plaque. CCTA can visualize the coronary artery lumen, even non‐obstructive atherosclerotic plaque and coronary artery stenosis severity. However, its application in the clinic is limited by the requirements of good image quality, extremely high spatial/temporal resolution and low heart rate (< 60 beats/minute).^3^ Therefore, new diagnostic strategies for AS must be investigated.

Inflammation caused by activated MΦ plays an important role in plaque vulnerability and has serious clinical consequences.^4^ It is important to image activated MΦ within plaque in vivo since early detection of vulnerable plaque would greatly improve prognosis of AS patients. AS noninvasive imaging has been proposed using ^99m^Tc‐anti‐vascular cell adhesion molecule‐1 (VCAM‐1) and ^18^F‐fludeoxyglucose (FDG). However, expression of VCAM‐1 on atherosclerotic plaque was too weak, whereas ^18^F‐FDG is not a specific tracer, and its use is hampered by vascular motion and high metabolic uptake in the myocardium.^2^, ^5^ So far, plaque imaging targeting MΦ has not been reported, and no suitable targets have been found for noninvasive AS imaging for early diagnose, which calls for an urgent search of novel molecules.

CD93 is a type 1 transmembrane glycoprotein that has pro‐inflammatory activity^6^, ^7^ and is highly expressed in MΦ and the endothelial cells during advanced inflammation. CD93 promotes adhesion, penetration and exudation of inflammatory cells to endothelium, and it has been postulated as an important inflammation marker.^8^, ^9^ Plaque rupture and thrombosis in AS is mainly due to inflammatory progression, where MΦ performs an important role. Indeed, accumulation of MΦ was proportional to plaque size.^10^, ^11^ The progression of AS is also closely related with accumulation of MΦ in the arterial wall.^12^, ^13^ Therefore, our hypothesis is that CD93 in MΦ may be used as a novel reporter in AS noninvasive plaque imaging. To test this, we prepared ^3^H‐2‐DG‐labelled CD93^hi^ and CD93^lo^ MΦ and a ^125^I‐α‐CD93 tracer (^125^I‐anti‐CD93mAb) to explore plaque‐chemotaxis in adoptive‐transferred CD93^hi^ and CD93^lo^ MΦ, as well as the targeting of ^125^I‐α‐CD93 to atherosclerotic plaques.

## 2 MATERIALS AND METHODS

### 2.1 Establishment of AS model in Sprague‐Dawley (SD) rats

All animal research protocols were approved by the Animal Care and Use Committee of Shandong University. The AS model was constructed as described previously.^14^, ^15^, ^16^, ^17^ Briefly, SD rats (male, 200 ± 20 g, Beijing Huafukang) were anaesthetized intraperitoneally with 0.6% phenobarbital sodium (1 ml/100 g), and the intima of the left carotid artery then damaged with a vascular clamp. The rats were fed with a high‐fat diet for four months and injected intraperitoneally with ovalbumin, bovine serum albumin and vitamin D_3_.^14^, ^18^, ^19^ To confirm the establishment of the model, a section of fresh embedded arteries was prepared for each group. These were stained with haematoxylin and eosin (H&E). The aorta was stained with Oil Red O Staining (Servicebio) to observe lipid droplets in plaque.

### 2.2 Preparation of ^3^H‐2‐DG‐labelled CD93^hi^ and CD93^lo^ MΦ

Preparation of CD93^hi^ MΦ: MΦ were isolated from the peritoneal cavity of naive SD rats with RPMI 1640 medium at 72 h post 6% potato starch intraperitoneal injection. After removal of non‐adherent cells after culture in a humidified incubator (37°C, 5% CO_2_) for 1 h, cells were stimulated with either Lipopolysaccharide (LPS, 0.1 μg/ml; Sigma) in RPMI‐10% foetal bovine serum, (Invitrogen, FBS) for 12 h (CD93 high expression, CD93^hi^), or without LPS stimulation (CD93 lower expression, CD93^lo^) as a control.

Expression of CD93: For reverse‐polymerase chain reaction (RT‐PCR), TRIZOL reagent (Invitrogen) was used to extract total RNA from MΦ to determine its concentration. cDNA was obtained through TransScript One‐Step gDNA Removal and cDNA Synthesis SuperMix (TRANSGENBIOTECH). Target DNA was separated by 1.5% agarose gel electrophoresis and analysed by Image J. CD93 primers were as follows: 5′‐TGCCCCACTCAAGATGCTG‐3′ (forward) and 5′‐CGCTTGCGATAGACCAGTAGC‐3′(reverse).

For Western blot, MΦ were lysed with RIPA lysate (Servicebio) to obtain a protein suspension. Total protein (20 μg) was subjected to gel electrophoresis (Bio‐Rad gel, Bio‐Rad Laboratories) at 80 mV for 30 min and then 120 mV for another 30 min. Total protein was transferred to a polyvinylidene fluoride at 200 mA for 90 min, followed by blocking by 5% skimmed milk powder for 2 h at room temperature. The antibodies used were (i) mouse anti‐mouse CD93 monoclonal antibody (1:200 dilution, Santa, America), (ii) rabbit anti‐mouse GAPDH polyclonal antibody (1:10 000 dilution, Abcam, UK) and (iii) goat anti‐mouse and goat anti‐rabbit secondary antibodies (1:10 000 dilution, Abcam, UK). The protein was quantitatively analysed with a Tanon 4200 imaging system (Tanon Science and Technology Co., Ltd., Shanghai, China).

Labelling with ^3^H‐2‐DG (2‐deoxyglucose): 40 MBq ^3^H‐2‐DG (Specific Activity, 0.74 TBq/mmol, China Isotope & Radiation Corporation) in RPMI 1640 medium was added into groups of CD93^hi^ and CD93^lo^ MΦ (1.5 × 10^8^ cells), and cultured at 37℃, 5% CO_2_ for 2 h. The supernatant was discarded, and the pellet was washed with PBS (phosphate buffered saline). Finally, MΦ were re‐suspended in 200 μl PBS and added to 4 ml of scintillation fluid (ULTIMA GOLD™). Radioactivity (cpm, counts per minute) was measured with a Wizardm1470 (PE) liquid scintillator. Ex vivo 72 h stability in RPMI 1640 and FBS was tested for ^3^H‐2‐DG‐labelled CD93^hi^ and CD93^lo^ MΦ. As mentioned above labelling with ^3^H‐2‐DG, 1 ml RPMI 1640 and FBS was added into the wells after washing with PBS and cells were collected after 72 h to measure radioactivity.

### 2.3 Effect of ^3^H‐2‐DG labelling on CD93^hi^ and CD93^lo^ MΦ cells

CD93^hi^ and CD93^lo^ MΦ were examined for CD93 and CD68 immunofluorescence staining. After labelling with ^3^H‐2‐DG, cell morphology was observed under the microscope and phagocytosis of Dil‐Ox‐LDL (Dil‐labelled oxidized low‐density lipoprotein) (20 μg/ml, Yiyuan Biotechnologies) by CD93^hi^ and CD93^lo^ MΦ was measured. Briefly, Dil‐Ox‐LDL was added to the cultured cells, at 37°C and 5% CO_2_ for 6 h. The supernatant was discarded, and cells were rinsed twice with PBS before addition of 100 μl 4′, 6‐diamidino‐2‐phenylindole (DAPI, Solarbio) and incubating at room temperature for 10 min and observation with an inverted fluorescence microscope. To test for cell apoptosis, CD93^hi^ and CD93^lo^ MΦ were re‐suspended in 500 μl 1 × binding buffer at 1 × 10^6^/ml. To these, 5 μl Annexin V‐FITC and 10 μl PI were added and incubated at room temperature for 5 min in the dark. To test for cell cycle, CD93^hi^ and CD93^lo^ MΦ were re‐suspended in 500 μl DNA staining solution containing 10 μl permeabilization solution (final concentration 1 × 10^6^/ml) and incubated for 30 min in the dark at room temperature. The samples were analysed by a flow cytometer (FACSCelesta), and data were analysed by FlowJo 7.6.

### 2.4 Preparation of ^125^I‐α‐CD93 and ^125^I‐IgG

Radioiodine125 labelling was performed with the Iodogen method. Briefly, 100 μg of α‐CD93 (anti‐CD93mAb, rabbit anti‐mouse monoclonal antibody, Bioss, America) and 38.5 MBq Na^125^I (China Isotope & Radiation Corporation, Specific Activity 13.6 GBq/ml) were incubated at room temperature for 20 min (100 μg of IgG (Solarbio) and 40.5 MBq Na^125^I were incubated in the same way). PD‐10 (GE Healthcare) was used for separation. The rate of labelling (the ratio of ^125^I‐α‐CD93 or ^125^I‐IgG to total input Na^125^I) and the radiochemical purity (the ratio of ^125^I‐α‐CD93 or ^125^I‐IgG to the total radioactivity) were determined by paper chromatography with a mobile phase of methanol/saline (v/v, 2:1). The tracers were diluted (1:20) in saline and serum at 24 h, 48 h and 72 h for stability analysis.

For the cell saturation assay, CD93^hi^ and CD93^lo^ MΦ cells were plated in a 6‐well plate (1 × 10^6^/well). In the total binding group, ^125^I‐α‐CD93 (1~80 nM in PBS) was added into the plates and incubated at 37°C for 2 h. In the non‐specific binding group, 300‐fold non‐labelled α‐CD93 was added 1 h before adding ^125^I‐α‐CD93. Finally, cells were collected and radioactivity was detected with a Gamma Counter (CII, WIPE TEST COUNTER, CPAINTEC). Maximum binding ability (*B*_max_) and dissociation constant (*K*_d_) were obtained using GraphPad Prism software. For the competitive binding assay, CD93^hi^ MΦ cells were plated in the same way as above using 0.00001~1000 nmol/L unlabelled α‐CD93 and 77 nmol/L ^125^I‐α‐CD93.

### 2.5 Phosphor autoradiography

For the ^3^H‐2‐DG‐labelled CD93^hi^ and CD93^lo^ MΦ adoptive transfer, phosphor autoradiography was performed in anaesthetized model rats 24 h, 48 h and 72 h after intraperitoneal injection of ^3^H‐2‐DG‐labelled CD93^hi^ and CD93^lo^ MΦ (3.7 MBq for each rat). Rats were laid on a Tritium sensitive screen (TR, PerkinElmer) for 1 h before scanning. After 72 h, the isolated aortic arch from a model rat was placed on the Tritium screen for 2 h and scanned. OptiQuant™ image analysis software 5.0 (PerkinElmer Life Sciences) was used to calculate the DLU/mm^2^ (Digital light units/mm^2^) for semi‐quantitative analysis.

For the groups of ^125^I‐α‐CD93 (3.7 MBq for each rat) or ^125^I‐IgG (3.7 MBq for each rat) injection, the iodine uptake of the thyroid gland was blocked by feeding water containing 4% sodium iodide (Meilunbio) 24 h before injection with ^125^I‐α‐CD93 or ^125^I‐IgG. Dynamic phosphor autoradiography was carried out at 24 h and 48 h after the injection of tracers. The left/right carotid artery and aorta were separated, and ex vivo autoradiography was performed after 48 h.

### 2.6 Ex vivo biodistribution

The serum, blood cells, bilateral carotid arteries, aorta and some other important organs (muscle, bone, kidney, thyroid, spleen, liver, intestine, heart and lung) in executed model rats were isolated 72 h after injection with ^3^H‐2‐DG‐labelled CD93^hi^ and CD93^lo^ MΦ cells. Samples were weighted and digested with 88% formic acid at 100 ℃ for 1 h, before decoloring with 200 µl of 30% hydrogen peroxide. Finally, samples were moved to a scintillation vial containing 4 ml of scintillation fluid (ULTIMA GOLD™). Radioactivity (β rays) was measured in the Liquid scintillator (MicroBeta TriLux, Perkin Elmer) after dark adaptation for 1 h. The ID (injected dose) and %ID/g (percentage injected dose per gram) were calculated relative to the injected dose (cpm), and the T/NT ratio (target/non‐target, left carotid artery/right carotid artery) was calculated.

In the ^125^I‐α‐CD93 and ^125^I‐IgG injection groups, the same tissue sample as above was collected 48 h after tracer injection. The tissue was weighted, and radioactivity (cpm, count per minute) was detected with a Gamma counter. The %ID/g (percentage injected dose per gram) was calculated according to the injected dose (cpm).

### 2.7 Immunohistochemical and Immunofluorescence staining

Immunohistochemical (IHC) and immunofluorescence (IF) staining of CD93 and CD68 in left and right carotid arteries and aorta were performed. Fresh tissues were fixed in 4% paraformaldehyde for 24 h, embedded in paraffin and cut into 4 μm thickness tissue sections. For IHC, CD68 and CD93 were stained with rabbit‐anti‐mouse CD68/CD93 mAbs (Servicebio, 1:500) and Horseradish Peroxidase (HRP)‐conjugated goat anti‐mouse secondary antibody (Servicebio, 1:200). Finally, diaminobenzidine (DAB) was added to produce a brown precipitate. For immunofluorescence staining, CD68 was stained with mouse‐anti‐mouse CD68 mAb (Servicebio, 1:500) and FITC (green)‐conjugated goat anti‐mouse IgG (Servicebio, 1:200). CD93 was stained with rabbit‐anti‐mouse CD93 polyclonal antibody (Bioss, 1:500) and Cy5 (red)‐conjugated goat anti‐rabbit IgG (Servicebio, 1:1000). Slides were observed under the general microscope or the fluorescence microscope at 400×. The Optic Density/Area (IOD/Area) in IHC was analysed by Image‐Pro Plus software 4.5.0.29 (Media Cybernetics).

### 2.8 Statistical analysis

Data analysis was performed with GraphPad Prism 9 software (GraphPad Software, Inc.). Each set of data was from at least three independent experiments. For general comparison, data were represented as the mean ± standard deviation and Student's *t*‐test was used. For T/NT ratios comparisons, median (interquartile range, IQR) and Wilcoxon rank sum test were used. The differences were considered significant when *p* < .05.

## 3 RESULTS

### 3.1 Validation of the AS model

**FIGURE 1:**
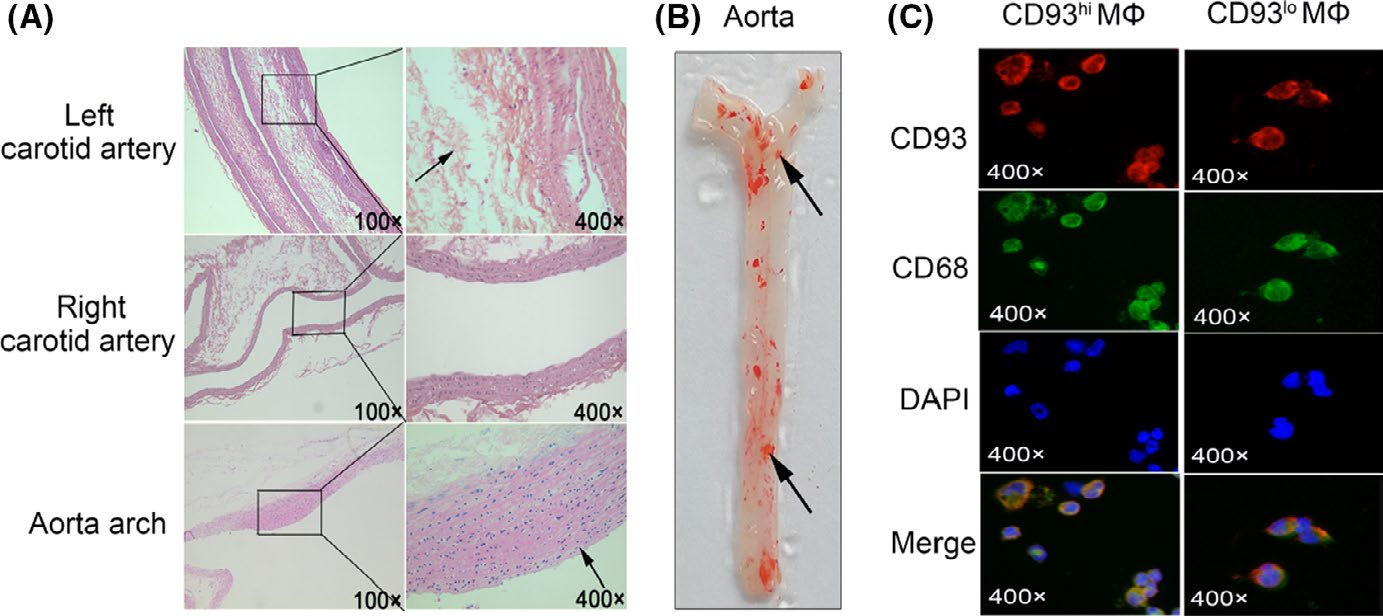
H&E and Oil red staining for rat artery and aorta. (A) Left/right carotid artery and aorta arch from the rat AS model, with representative H&E staining images at 100× and 400× magnification. The black arrow points to the atherosclerotic plaque in the carotid artery (left) and the aorta. (B) Oil red O staining of aorta in the AS model, with black arrow pointing to lipid droplets. (C) Immunofluorescence staining of CD93^hi^ and CD93^lo^ MΦ and representative images (400×), CD93 (red), CD68 (green) and nucleus (blue). Data were obtained from three independent experiments

The H&E staining in the established AS model rats indicated that atherosclerotic plaques were formed in the clamped left carotid artery and in the aortic arch (Figure 1A). Oil red staining showed a clear lipid deposition in the aorta (Figure 1B). These results suggest that the AS model was successfully established.

### 3.2 Effect of LPS stimulation on MΦ

**FIGURE 2:**
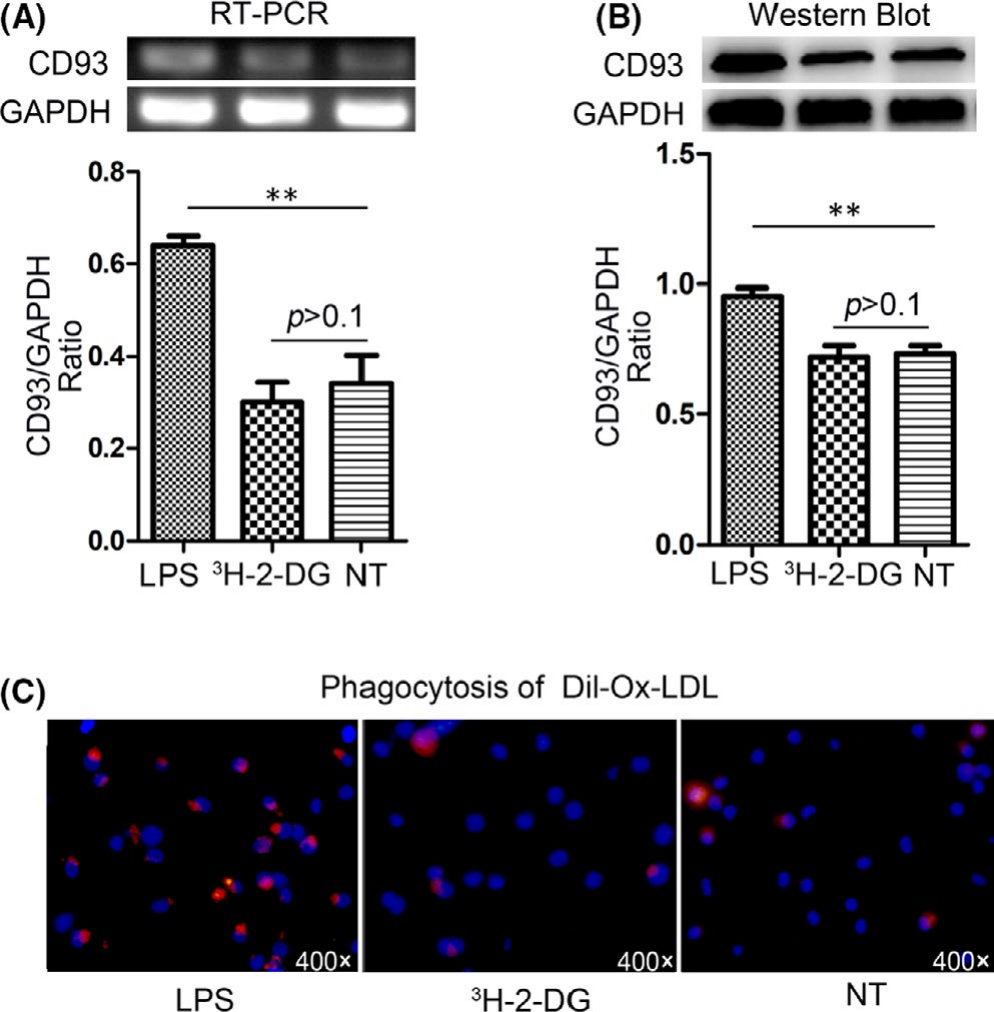
Effect of LPS stimulation and ^3^H‐2‐DG labelling on CD93 expression and MΦ function. (A) CD93 mRNA expression after LPS stimulation and ^3^H‐2‐DG labelling was detected by RT‐PCR. (B) CD93 protein expression after LPS stimulation and ^3^H‐2‐DG labelling was detected by Western Blot. (C) Phagocytosis of Dil‐Ox‐LDL by MΦ (400×), with Dil‐Ox‐LDL (red) and nuclear (blue). NT: non treated by LPS, no ^3^H‐2‐DG labelling, LPS: LPS‐stimulated, ^3^H‐2‐DG: labelled with ^3^H‐2‐DG. Data were analysed by Student's *t*‐test (*n* = 3), **p < .01

**FIGURE 3:**
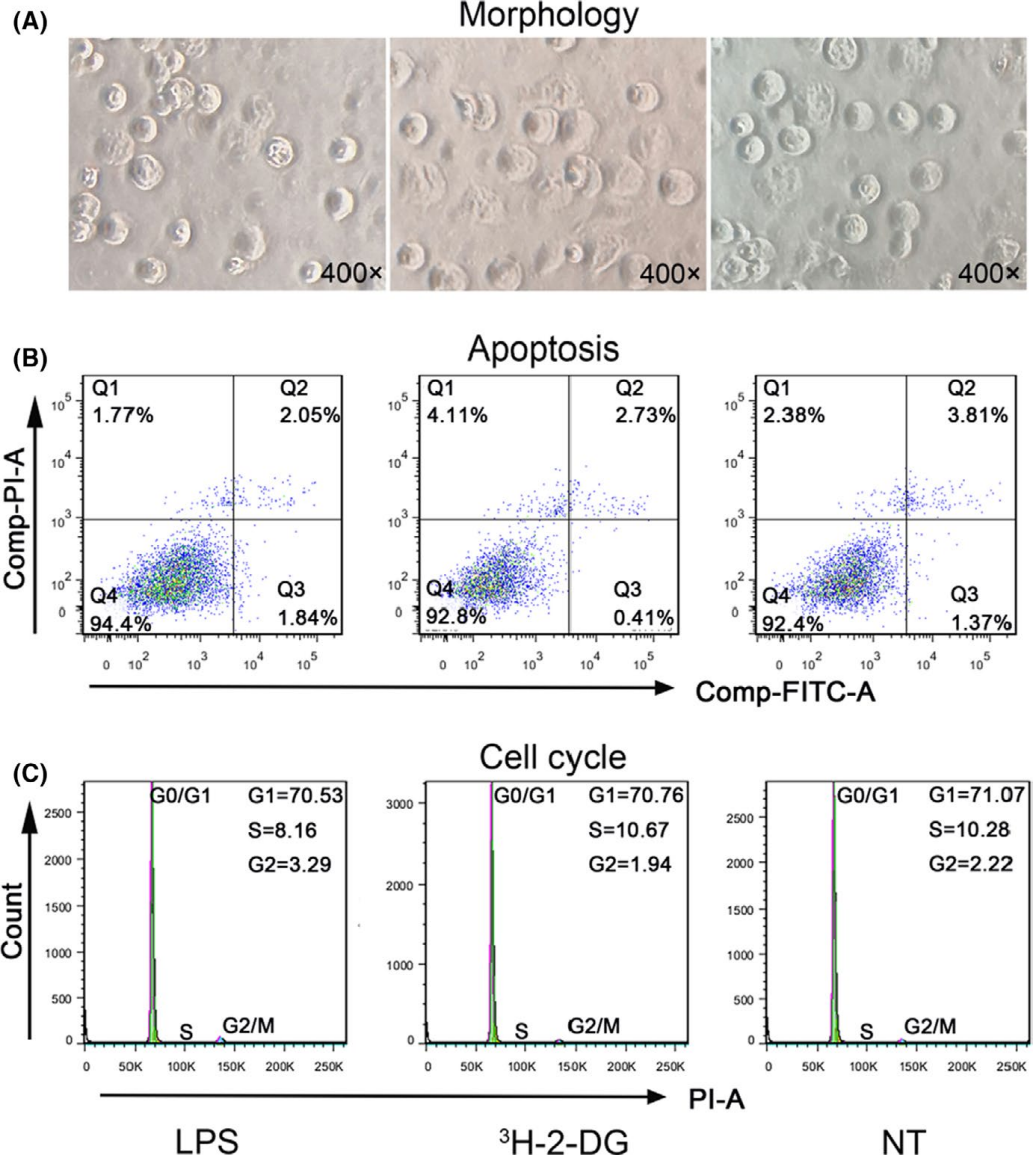
Effect of LPS stimulation and ^3^H‐2‐DG labelling on CD93 expression and MΦ function. (A) Morphology (400×) after LPS stimulation and ^3^H‐2‐DG labelling. (B) Apoptosis of MΦ detected by flow cytometry. (C) Cell cycle of MΦ detected by flow cytometry. G1: pre‐DNA synthesis stage, NT: non treated by LPS, no ^3^H‐2‐DG labelling, LPS: LPS‐stimulated, ^3^H‐2‐DG: labelled with ^3^H‐2‐DG. Data were obtained from three independent experiments

Immunofluorescence staining confirmed that CD93 and CD68 were co‐expressed in MΦ (Figure 1C). After LPS stimulation, expression of CD93 in MΦ increased at both mRNA and protein levels (*p* < .01; *p* < .05 respectively), in contrast to those with no LPS stimulation (Figure 2A,B). More interestingly, after LPS stimulation, phagocytosis of Dil‐Ox‐LDL by MΦ was significantly enhanced compared to those non‐stimulated (*p* < .05) (Figure 2C). In contrast, LPS stimulation had no effect on MΦ morphology (Figure 3A), apoptosis (living cell ratios of 94.4% and 92.4% in LPS‐stimulated and control groups respectively) (Figure 3B) or cell cycle (G1 phase ratio of 70.53% and 71.07% in LPS‐stimulated and control groups respectively) (Figure 3C).

### 3.3 Successful preparation of ^3^H‐2‐DG‐labelled CD93^hi^ and CD93^lo^ MΦ cells

Two hours after incorporation of ^3^H‐2‐DG, radioactivity of ^3^H‐2‐DG‐labelled CD93^hi^ and CD93^lo^ MΦ reached 45739 ± 1669 and 34807 ± 510 cpm respectively. Importantly, after 72 h radioactivity of ^3^H‐2‐DG‐labelled CD93^hi^ and CD93^lo^ MΦ was still unchanged (*p* > .05): 42904 ± 176 and 33655 ± 15 in RPMI 1640; 43038 ± 35 and 34246 ± 27 in FBS. This indicates that ^3^H‐2‐DG‐labelled CD93^hi^ and CD93^lo^ MΦ is stable for at least 72 h in both RPMI 1640 and FBS. Further, compared to unlabelled cells, ^3^H‐2‐DG‐labelled CD93^hi^ and CD93^lo^ MΦ showed no changes in CD93 expression (*p* > .05) (Figure 2A,B), phagocytosis of Dil‐Ox‐LDL (Figure 2C), morphology (as observed under the microscope) (Figure 3A), apoptosis (measured through flow cytometry, living cell ratio 92.4% vs 92.8% respectively) (Figure 3B) or cell cycle (G1 phase ratio 71.07% vs 70.76% respectively) (Figure 3C).

### 3.4 Preparation of ^125^I‐α‐CD93 and ^125^I‐IgG

^125^I‐α‐CD93 and ^125^I‐IgG were successfully prepared, with labelling rates of 97.01 ± 0.72% and 91.79 ± 0.64% respectively. The radiochemical purity of both tracers was higher than 96%, and was still 90% in normal saline and rat serum even after 72 h.

**FIGURE 4:**
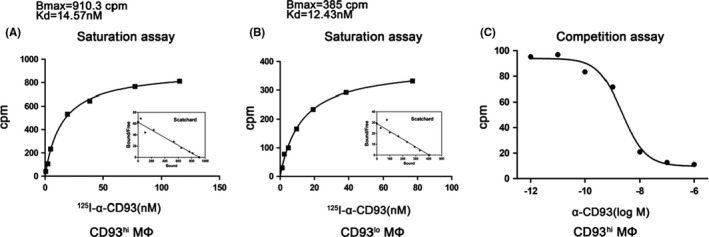
Evaluation of radiolabelled tracers. (A) Representative saturation binding curve and Scatchard plots with increasing ^125^I‐α‐CD93 binding to CD93^hi^ MΦ; (B) the same for CD93^lo^ MΦ. (C) Competition binding curve between ^125^I‐α‐CD93 and increasing unlabelled α‐CD93, *n* = 5

Saturation binding assays for CD93^hi^ MΦ and CD93^lo^ MΦ cells showed a similar affinity with *K*_d_ (nM) of 14.57 vs 12.43, respectively, but total CD93 molecules increased in CD93^hi^ MΦ with *B*_max_ of 910.30 vs 385 (cpm/10^4^ cells), respectively (Figure 4). Importantly, addition of 300‐fold unlabelled α‐CD93 could block the binding of ^125^I‐α‐CD93 to CD93^hi^ MΦ almost completely, with *K*_i_ value of 2.19 nM, further confirming the specific binding between ^125^I‐α‐CD93 and CD93.

### 3.5 Phosphor autoradiography of ^3^H‐2‐DG‐labelled CD93^hi^ and CD93^lo^ MΦ adoptive transfer groups

**FIGURE 5:**
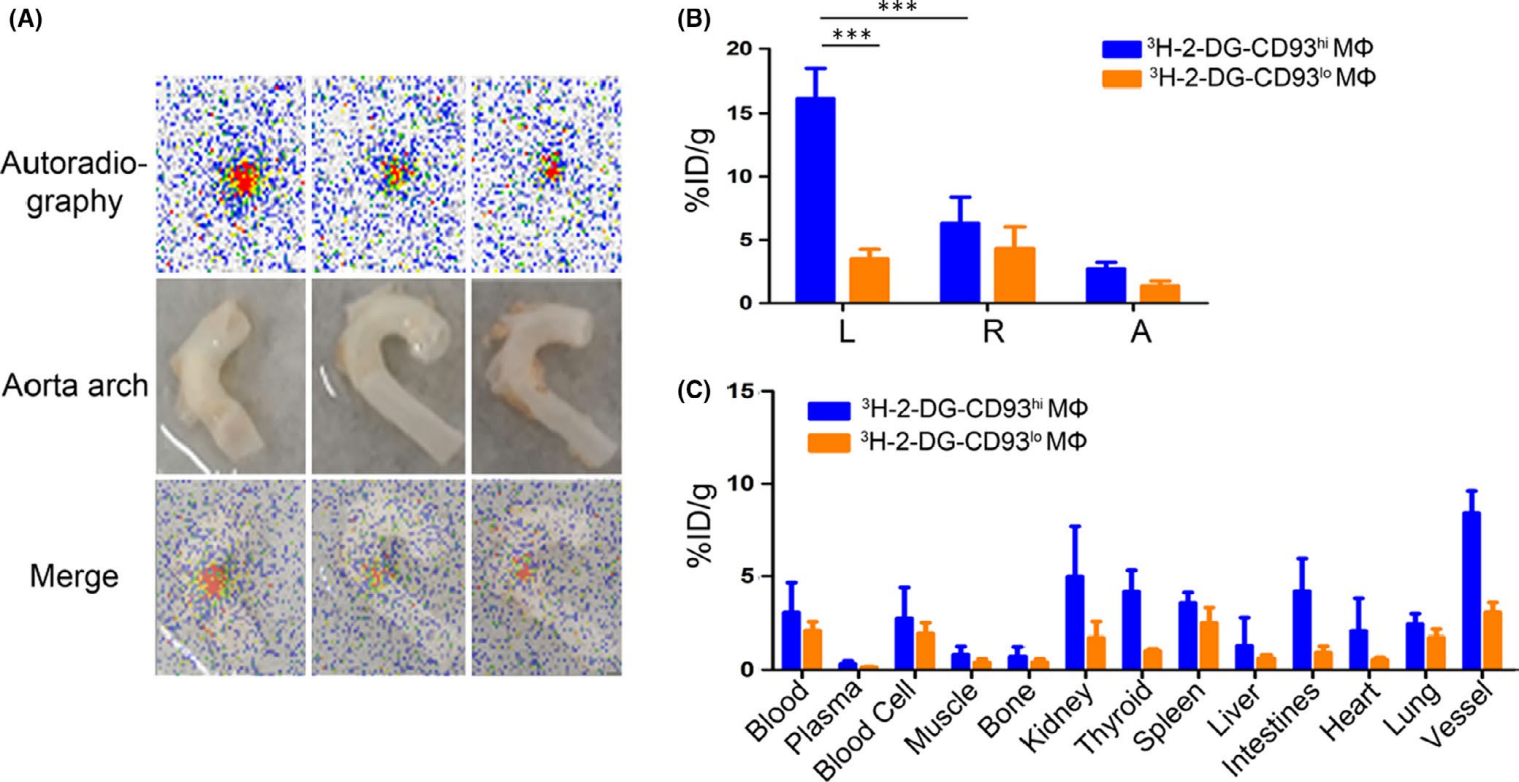
Tritium‐phosphor autoradiography and biodistribution of ex vivo aorta arch 72 h after ^3^H‐2‐DG‐labelled CD93^hi^ and CD93^lo^ MΦ adoptive transfer. (A) Representative ex vivo tritium‐phosphor autoradiography images of aortic arch in AS model after 72 h; (B/C) radioactivity biodistribution (%ID/g) of representative tissues in the ^3^H‐2‐DG‐labelled CD93^hi^ and CD93^lo^ MΦ adoptive transfer group after 72 h, with L (left carotid artery), R (right carotid artery) and A (aortic arch). *n* = 5, *** *p* < .001

No apparent radioactivity accumulation was observed in dynamic whole‐body autoradiography with Tritium screen scanned 24 h, 48 h or 72 h after ^3^H‐2‐DG‐labelled CD93^hi^ and CD93^lo^ MΦ adoptive transfer (not shown). In contrast, radioactivity accumulated in the aorta arch after 72 h in an ex vivo autoradiography screen in the ^3^H‐2‐DG‐CD93^hi^ MΦ adoptive transfer group (Figure 5A). No obvious radioactivity accumulated in the ^3^H‐2‐DG‐CD93^lo^ MΦ adoptive transfer group (Figure S1A), indicating that CD93^hi^ MΦ was highly chemotactic for the AS plaque. Also, no radioactivity accumulation was observed in the ^3^H‐2‐DG injection control group (Figure S1B), suggesting specific binding between the AS plaque and ^3^H‐2‐DG‐CD93^hi^ MΦ.

### 3.6 Biodistribution for ^3^H‐2‐DG‐labelled CD93^hi^ and CD93^lo^ MΦ adoptive transfer groups

Radioactivity in the three tested arteries increased with time (Figure S2A). After 72 h, the accumulated radioactivity (%ID/g, ^3^H‐2‐DG‐CD93^hi^ MΦ) was higher in the clamped left carotid artery than in the non‐clamped right carotid artery (*p* < .001) and in the aorta (*p* < .001) (Figure 5B), indicating severe inflammatory reactions caused by CD93^hi^ MΦ infiltration and over‐expression of CD93 in damaged left carotid artery and aorta. The T/NT ratios for the ^3^H‐2‐DG‐CD93^hi^ MΦ adoptive transfer group after 24, 48 and 72 h were 1.41 (0.44), 1.40 (0.32) and 2.63 (0.49) (24 h vs 72 h, *p* < .05; 48 h vs 72 h, *p* < .05). After 72 h, the uptake (%ID/g) of ^3^H‐2‐DG‐CD93^lo^ MΦ in the above three arteries was 3.52 ± 0.74, 4.31 ± 1.73, 1.37 ± 0.40 respectively (Figure 5B). The uptake of ^3^H‐2‐DG (Figure S2B) in the above three arteries was only 1.91 ± 0.47, 1.99 ± 0.26, 2.03 ± 0.53 (%ID/g) respectively. Compared with the ^3^H‐2‐DG‐CD93^lo^ MΦ adoptive transfer and ^3^H‐2‐DG groups, the ^3^H‐2‐DG‐CD93^hi^ MΦ adoptive transfer group exhibited a stronger AS plaque chemotactic tendency in the clamped left carotid artery (*p* < .001, *p* < .001 respectively). The T/NT ratio of the ^3^H‐2‐DG‐CD93^hi^ MΦ group was higher than in the ^3^H‐2‐DG‐CD93^lo^ MΦ group (0.68 (0.06)) (*p* < .05) and in the ^3^H‐2‐DG group (0.96 (0.09)) (*p* < .05). In the ^3^H‐2‐DG‐CD93^hi^ MΦ adoptive transfer group, except for plaque tissue, higher radioactivity (%ID/g) were also detected in liver, kidney, spleen, lung and intestine, which indicated that the probe was mainly metabolized through the renal pathway (Figure 5C).

### 3.7 Phosphor autoradiography for ^125^I‐α‐CD93 and ^125^I‐IgG injection groups

**FIGURE 6:**
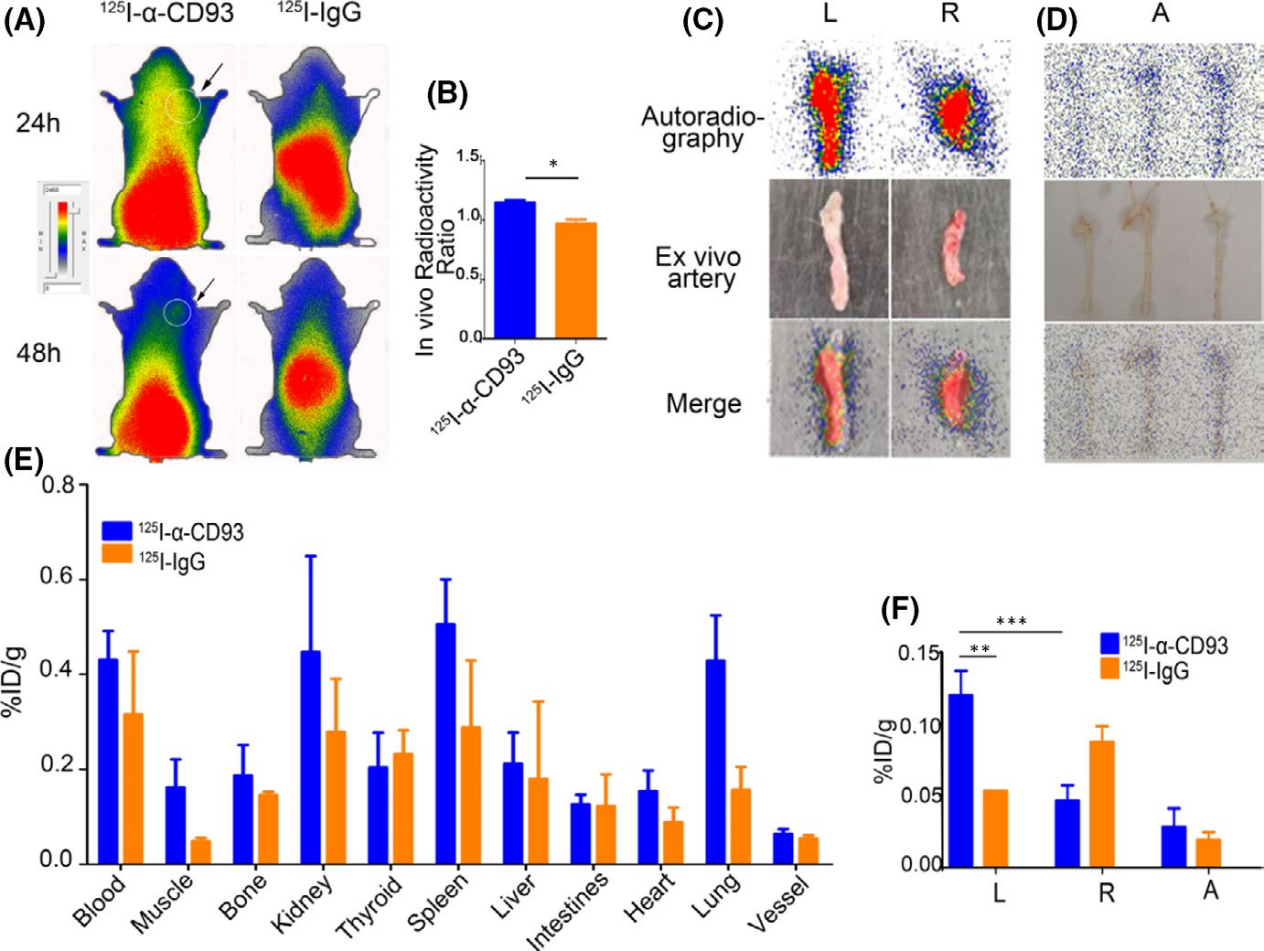
Whole‐body and ex vivo artery phosphor autoradiography and biodistribution. (A) Representative images of ^125^I‐α‐CD93 and ^125^I‐IgG in AS model whole‐body phosphor autoradiography after 24 and 48 h, with black arrow pointing to the left carotid artery. (B) In vivo radioactivity ratio (left carotid artery/right carotid artery) between ^125^I‐α‐CD93 and ^125^I‐IgG; representative images of ex vivo (C) left/right carotid artery and (D) aorta artery after 48 h. (E) Biodistribution of ^125^I‐α‐CD93 and ^125^I‐IgG after 48 h. (F) Biodistribution of ^125^I‐α‐CD93 and ^125^I‐IgG in left/right carotid artery and aorta after 48 h. L (left carotid artery), R (right carotid artery) and A (aorta). Data were obtained from three independent experiments (*n* = 5), * *p* < .05, ** *p* < .01, *** *p* < .001

Dynamic whole‐body phosphor autoradiography was performed 24 h and 48 h post‐injection with ^125^I‐α‐CD93 and ^125^I‐IgG. After ^125^I‐α‐CD93 injection, radioactivity accumulated in the clamped left carotid artery after 48 h, in contrast with the right carotid artery. Radioactivity (DLU/mm^2^) of the clamped left carotid artery reached 682230 ± 90662, but only 424275.50 ± 2253.50 in the right carotid artery. No obvious radioimage was observed after ^125^I‐IgG injection at 24 h or 48 h, indicating that accumulation of ^125^I‐α‐CD93 in the carotid artery was specific (Figure 6A). In vivo radioactivity ratio between left and right carotid artery (DLU/DLU) for the ^125^I‐α‐CD93 and ^125^I‐IgG groups was 1.14 ± 0.03 and 0.97 ± 0.03 (*p* < .05) (Figure 6B).

Ex vivo carotid artery phosphor autoradiography imaging showed higher radioactivity in the left clamped carotid artery with plaque than in the non‐damaged right carotid artery (Figure 6C). The aorta arch showed obvious radioactivity accumulation in ^125^I‐α‐CD93 group compared with ^125^I‐IgG group (^125^I‐IgG group data were not shown) (Figure 6D), consistent with the results of whole‐body phosphor autoradiography.

### 3.8 Biodistribution of ^125^I‐α‐CD93 and ^125^I‐IgG injection groups

^125^I‐α‐CD93 accumulated in vivo in the kidney, spleen and lung (Figure 6E). Accumulation of ^125^I‐α‐CD93 (%ID/g) in the left carotid artery 48 h after ^125^I‐α‐CD93 injection was higher than in the right carotid artery (*p* < .001) and aorta (*p* < .001) (Figure 6F). Compared with the ^125^I‐IgG group, the left carotid artery in the ^125^I‐α‐CD93 group had obvious radioactivity accumulation (*p* < .005) (Figure 6F). The T/NT ratio after ^125^I‐α‐CD93 injection was higher than after ^125^I‐IgG injection (2.63 (0.23) vs 0.66 (0.02), *p* < .01).

### 3.9 Immunofluorescence and Immunohistochemistry staining

**FIGURE 7:**
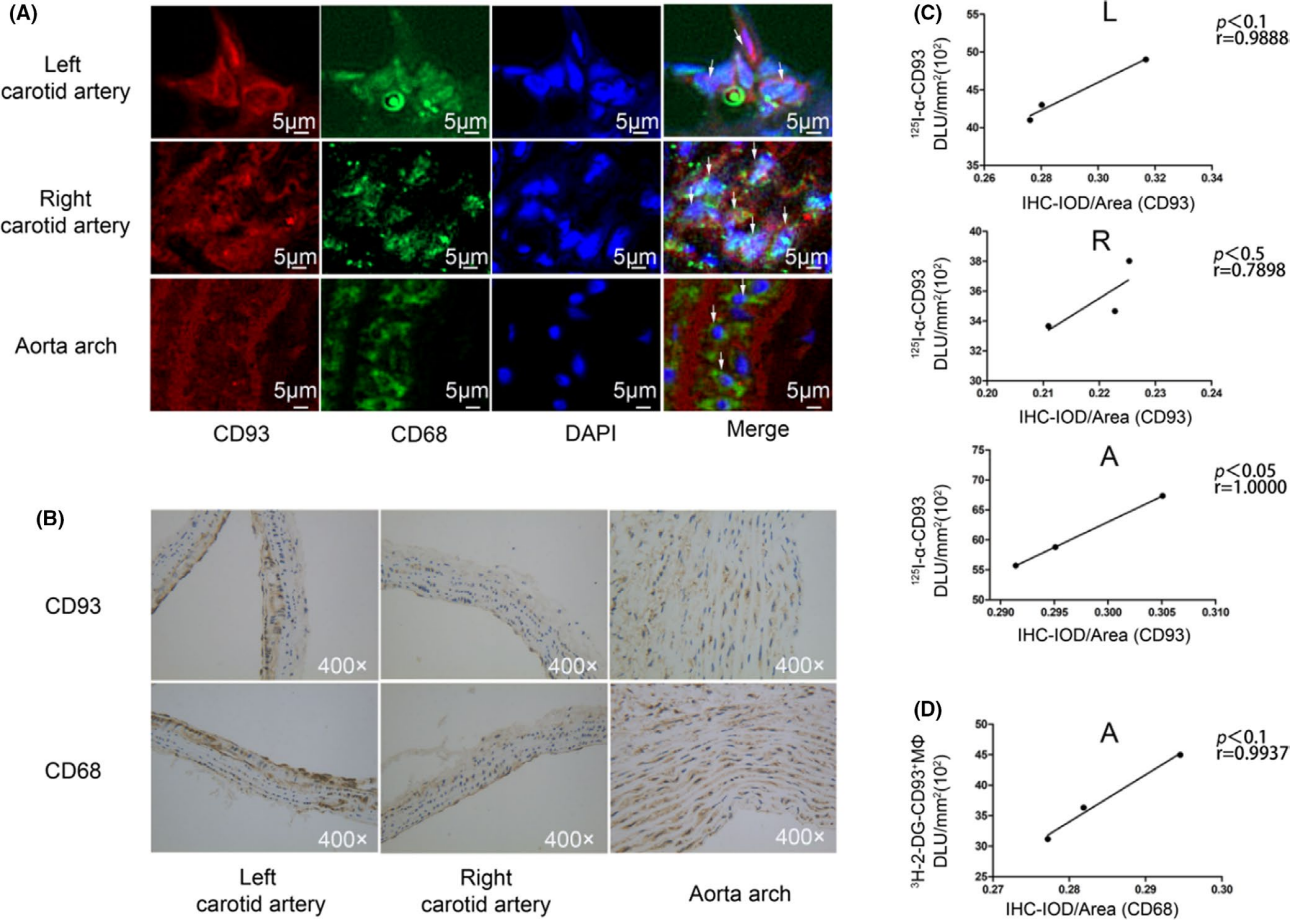
Immunofluorescence and immunohistochemistry imaging of diseased vessels. (A) Immunofluorescence staining of nucleus with DAPI (blue), CD68 (green) and CD93 (red). (B) Immunohistochemistry staining (400×) of CD93 and CD68, with positive staining shown as brown colour. (C) Correlation between IOD/Area of CD93 in immunohistochemistry staining and ^125^I‐α‐CD93 (DLU/mm^2^, digital light units) in ex vivo artery phosphor autoradiography. (D) Correlation between IOD/Area of CD68 in immunohistochemistry staining and ^3^H‐2‐DG‐CD93^hi^ MΦ in ex vivo aorta tritum‐phosphor autoradiography. L (left carotid artery), R (right carotid artery) and A (aorta). *n* = 3

To further confirm that the adoptive‐transferred MΦ was infiltrated and that CD93 was over‐expressed in AS plaque, we performed CD68 and CD93 double staining ex vivo. More CD68/CD93 were co‐expressed in the left carotid artery and in the aorta arch, suggesting that ^3^H‐2‐DG‐labelled CD93^hi^ MΦ exhibited higher plaque chemotactic tendency (Figure 7A,B). No obvious infiltration of CD93^hi^ MΦ was observed in the right carotid artery. There was a positive correlation between CD93 expression (IHC staining) and radioactivity accumulation (^125^I‐α‐CD93 ex vivo phosphor autoradiography) at the left carotid artery (*p* < .1, *r* = .99), aortic arch (*p* < .05, *r* = 1.00) and right carotid artery (*p* < .5, *r* = .79) (Figure 7C). Also, CD68 expression (IHC staining) and radioactivity (^3^H‐2‐DG‐CD93^hi^ MΦ ex vivo phosphor autoradiography) in the aorta arch were positively correlated (*p* < .1*, r* = .99), indicating that more CD93^hi^ MΦ moved to the aorta arch (Figure 7D).

## 4 DISCUSSION

Vulnerable plaque is a key factor in the development of coronary artery diseases (CADs). Early diagnosis, especially by noninvasive plaque imaging, is particularly important for prognosis. Current diagnostic modalities have limitations. For example, intravascular ultrasound is useful to evaluate the size, distribution and characteristics of the plaque, but relies on having sufficient operator parameters and good spatial resolution^20^; another method, CCTA, can classify the type of calcified high‐risk plaque, but requires high‐resolution imaging.^21^ Finally, MRI provides information, about wall geometry and plaque pressure, but it is not cost‐effective.^21^ Overall, these methods show anatomical changes in the advanced plaque stage and therefore do not efficiently detect early AS. Molecular imaging, in contrast, is independent from anatomical and morphological considerations and can be used for early and real‐time monitoring by using specific molecular targeting.^22^ However, successful noninvasive molecular imaging requires finding new molecular targets that have high selectivity and specificity.

Anti‐inflammatory therapy with Canakinumab (targeting interleukin‐1β) reduced the incidence of cardiovascular disease. Considering that inflammation plays an indispensable role in the development of vulnerable plaques, molecular imaging may be complementary to current anatomical imaging.^23^, ^24^ MΦ cells play a pivotal role in development of AS. They are the main inflammatory cells infiltrated into the arterial intima^25^ and phagocytize a large amount of oxidized lipids. They are then transformed into foam cells, playing crucial roles in the early stage of atherosclerotic lesions.^12^ MΦ in advanced lesions shows deficiency in the clearing of apoptotic cells, whereas post‐apoptotic necrotic cells release inflammatory mediators that can lead to plaque deterioration. Furthermore, MΦ participates in plaque rupture or internal haemorrhage through secretion of proteases.^25^

CD93 is expressed in a variety of inflammatory diseases, and is related to severity and prognosis. CD93 promotes the adhesion, penetration and exudation of inflammatory cells. For example, in patients with neovascular age‐related muscular degeneration (AMD), both transmembrane and soluble CD93 (sCD93) are over‐expressed.^26^ Inflammation triggers the in vivo release of sCD93 derived from inflammatory MΦ cells. A significant positive correlation exists between plasma sCD93 levels and the incidence of MI (myocardial infarction) / CADs: in a retrospective study of 120 acute MI patients, sCD93 in blood was elevated, correlating with poor clinical prognosis.^27^

At present, tracing of cell adoption has been widely used, such as inflammatory corpuscle, MΦ and inflammatory factor. Adoptive transfer of natural or genetically modified cytotoxic T cells have been used for selective immunotherapy against tumours.^28^, ^29^ Donor spleen cell infusion induced a stable immune tolerance in a miniature pig lung transplantation model.^30^ ^18^F‐FDG‐labelled splenocytes have been used in vivo to track cell aggregation in skin allograft.^31^ Since both CD93 and MΦ play a pivotal role in AS, we hypothesize that CD93 in MΦ may be a novel biomarker for AS plaque. Adoptive‐transferred MΦ in mouse requires 72 h for complete distribution into different organs.^32^ Herein, ^3^H‐2‐DG‐labelled CD93^hi^ MΦ were adoptedly transferred into an AS rat model. We traced these MΦ with super‐sensitive tritium‐phosphor autoradiography after 72 h. No radioactivity accumulation was detected in the diseased vessel in whole‐body tritium‐phosphor autoradiography, but it was detected ex vivo. In the same model, the left carotid artery (plaque formed) showed higher radioactivity accumulation than the right carotid artery (without plaque). Also, the signal for ^3^H‐2‐DG‐CD93^hi^ MΦ is higher as compared to ^3^H‐2‐DG‐CD93^lo^ MΦ in many organs which may be caused by CD93^hi^ macrophage more easily infiltrating into endothelium and epithelium, staying in this organ longer than CD93^lo^ macrophage. We would search for underlying mechanism of these high distribution organs in the future. To investigate whether CD93 is a target for AS plaque, we successfully prepared a high affinity ^125^I‐α‐CD93 tracer. Whole‐body/Ex vivo artery phosphor autoradiography and biodistribution with ^125^I‐α‐CD93 strongly indicated higher radioactivity accumulated in the clamped left carotid artery than in the right carotid artery. This not only was observed for the ^3^H‐2‐DG‐CD93^hi^ MΦ adoptive‐transferred group, but also for the inflammation‐caused high expression of CD93, suggesting that CD93 is a potential new reporter for AS noninvasive molecular imaging.

That labelled markers on MΦ can be used in vivo to trace their migration, and chemotactics has been suggested before. Radionuclide‐labelled specific molecular markers in MΦ in vivo have been used to trace MΦ through SPECT (single‐photon emission computed tomography)/CT imaging, for example folate receptor‐β (FR‐β),^4^ F4/80 receptor^33^ or macrophage mannose receptor (MMR).^34^ In our study, we prepared ^3^H‐2‐DG‐CD93^hi^ MΦ with high affinity and stability to trace MΦ chemotactic tendency to AS plaque with tritium screen autoradiography detection 72 h after MΦ adoptive transfer. We labelled MΦ with ^3^H‐2‐DG because 2‐DG, a glucose analog, is ingested by cells without being metabolized. This allows ex vivo observation of radiolabelled MΦ distribution. Labelled MΦ chemotaxis to AS plaque was also investigated.^5^, ^25^ Our data suggest that CD93, a biomarker of activated MΦ, has better specificity for imaging vulnerable plaques.

^18^F‐FDG, widely used in the clinic, as a non‐specific radiotracer, can be taken up by any cell that metabolizes glucose. Therefore, the myocardium tends to have a higher signal to weaken the imaging of the plaque.^24^, ^35^ High selective and specific tracers also have been used for plaque imaging studies, such as ^99m^Tc (^99m^Technetium)‐hydrazinonicotinamide (HYNIC)‐IL‐2,^36^ ^99m^Tc‐Annexin V and ^99m^Tc‐Duramycin.^37^ A nanobody‐tracer that targeted the MMR had non‐negligible background levels due to the presence of MMR‐positive macrophages lining the vascular adventitia.^34^ ^99m^Tc‐scFv‐VCAM‐1, a single‐chain variable fragment (scFv) of VCAM1 labelled with ^99m^Tc, showed low sensitivity in the detection of vulnerable plaque through SPECT.^2^ ScFv represents the idiotype of an antibody and is one of the smallest antibody fragments that retain the antigen‐binding specificity. They can be genetically engineered to enhance scFv antigen‐binding activity, removed non‐specific competitive surface proteins to make the background clearer.^38^ However, due to the lower binding affinity and fast blood clearance of scFv as an imaging probe, it was unfavourable for delay imaging study.^39^ Targeted radioimmunoimaging with monoclonal antibodies has become a reliable strategy.^40^ Our results suggest that in vivo radionuclide imaging with a ^125^I‐α‐CD93 tracer could be used as a new strategy to evaluate vulnerable plaques.

Our study only used ex vivo diseased vessel autoradiography imaging due to the low energy and tracing dose of tritium. However, biodistribution studies in same model confirmed much more radioactivities in diseased vessels. In future experiments, other suitable higher‐energy radionuclides can be tested to label macrophages and small peptides or antibody fragment of α‐CD93 can be labelled.

In conclusion, our results suggest that CD93^hi^ MΦ adoptively transferred have a high chemotactic tendency to plaques, where CD93 is highly expressed. Both ^3^H‐2‐DG‐labelled CD93^hi^ MΦ and ^125^I‐α‐CD93 radiotracer could target CD93 on the atherosclerotic plaque. CD93 in MΦ may be a potential new reporter for noninvasive atherosclerotic plaque imaging.

## CONFLICT OF INTEREST

The author declares that there is no conflict of interest.

## AUTHOR CONTRIBUTIONS

**Chen Su:** Data curation (equal); Formal analysis (equal); Writing—original draft (equal). **Yeming Han:** Investigation (equal); Methodology (equal); Writing—original draft (equal). **Bin Qu:** Data curation (equal); Writing—original draft (equal). **Chao Zhang:** Data curation (equal); Formal analysis (equal); Investigation (equal). **Ting Liang:** Formal analysis (equal); Writing—review & editing (equal). **Feng Gao:** Investigation (equal); Writing—review & editing (equal). **Guihua Hou:** Conceptualization (equal); Funding acquisition (equal); Resources (equal); Writing—review & editing (equal).

## Supporting information

Fig S1‐S2

Supplementary Material

## Data Availability

The data sets generated and/or analysed during the current study are available from the corresponding author upon reasonable request.
