# Supplementary material for: CD93 in macrophages: A novel target for atherosclerotic plaque imaging?
Source: J Cell Mol Med. 2022 Feb 15;26(8):2152–62. doi: 10.1111/jcmm.17237 (PMC8995462; doi:10.1111/jcmm.17237)
Supplement: Supplementary file 1 — Fig S1‐S2 [file JCMM-26-2152-s002.docx]

**
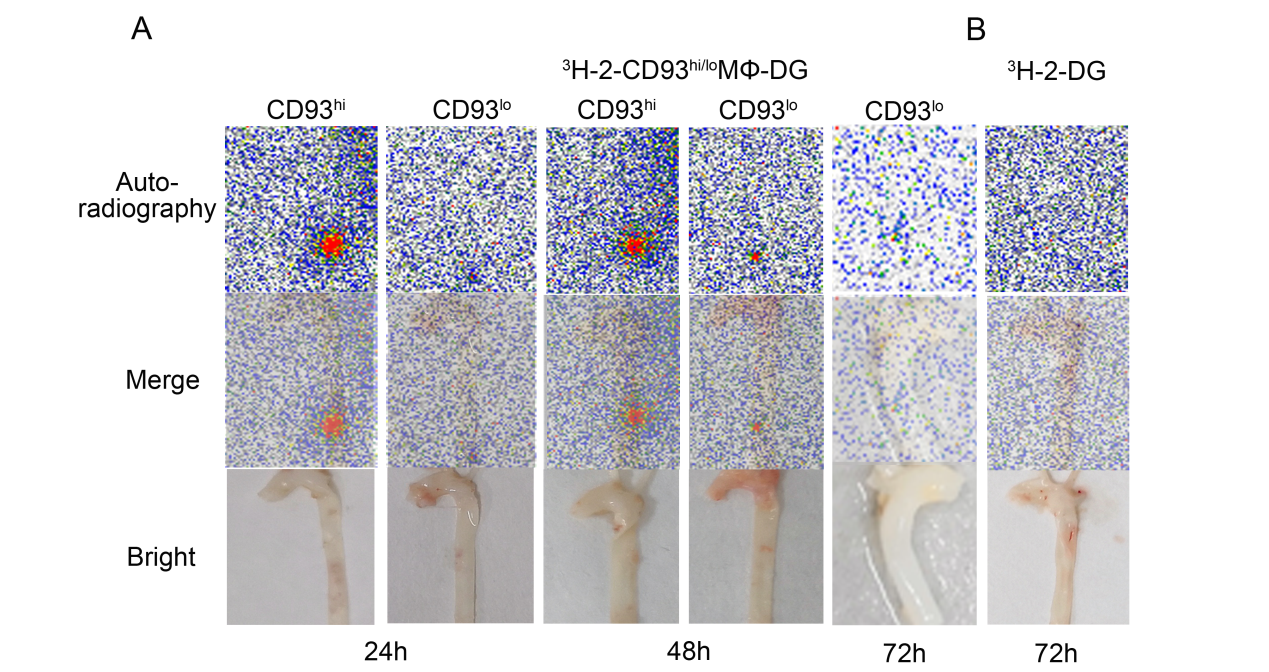
**

**Supplementary Figure 1** Tritium-phosphor-autoradiography of *ex vivo* aorta arch after ^3^H-2-DG labeled CD93^hi^ and CD93^lo^ MΦ and ^3^H-2-DG adoptive

(A) Representative *ex vivo* tritium-phosphor-autoradiography images of ^3^H-2-DG labeled CD93^hi^ and CD93^lo^ MΦ in aortic arch after 24, 48 and 72 h. (B) Representative *ex vivo* tritium-phosphor-autoradiography images of  ^3^H-2-DG in aortic arch after 72 h. n = 5

**
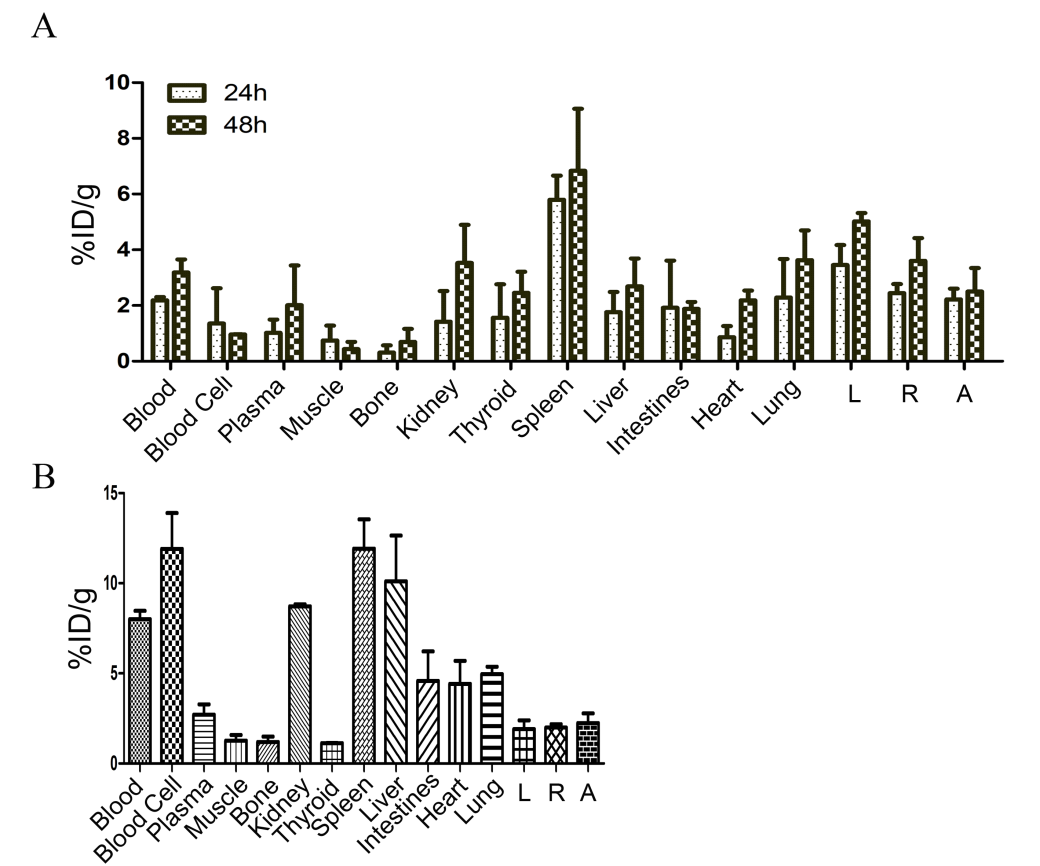
**

**Supplementary Figure 2.** *Ex-vivo* biodistribution after ^3^H-2-DG labeled CD93^hi^ and CD93^lo^ MΦ and ^3^H-2-DG adoption

(A) Radioactivity biodistribution of representative tissues in the ^3^H-2-DG labeled CD93^hi^ and CD93^lo^MΦ adoption group after 24 and 48 h. (B) Biodistribution of representative tissues in the ^3^H-2-DG adoption group after 72 h, L (left carotid artery), R (right carotid artery), A (aortic arch) n=5
