# Supplementary material for: CD93 in macrophages: A novel target for atherosclerotic plaque imaging?
Source: J Cell Mol Med. 2022 Feb 15;26(8):2152–62. doi: 10.1111/jcmm.17237 (PMC8995462; doi:10.1111/jcmm.17237)

1. Figure1A, Left carotid artery (200×):


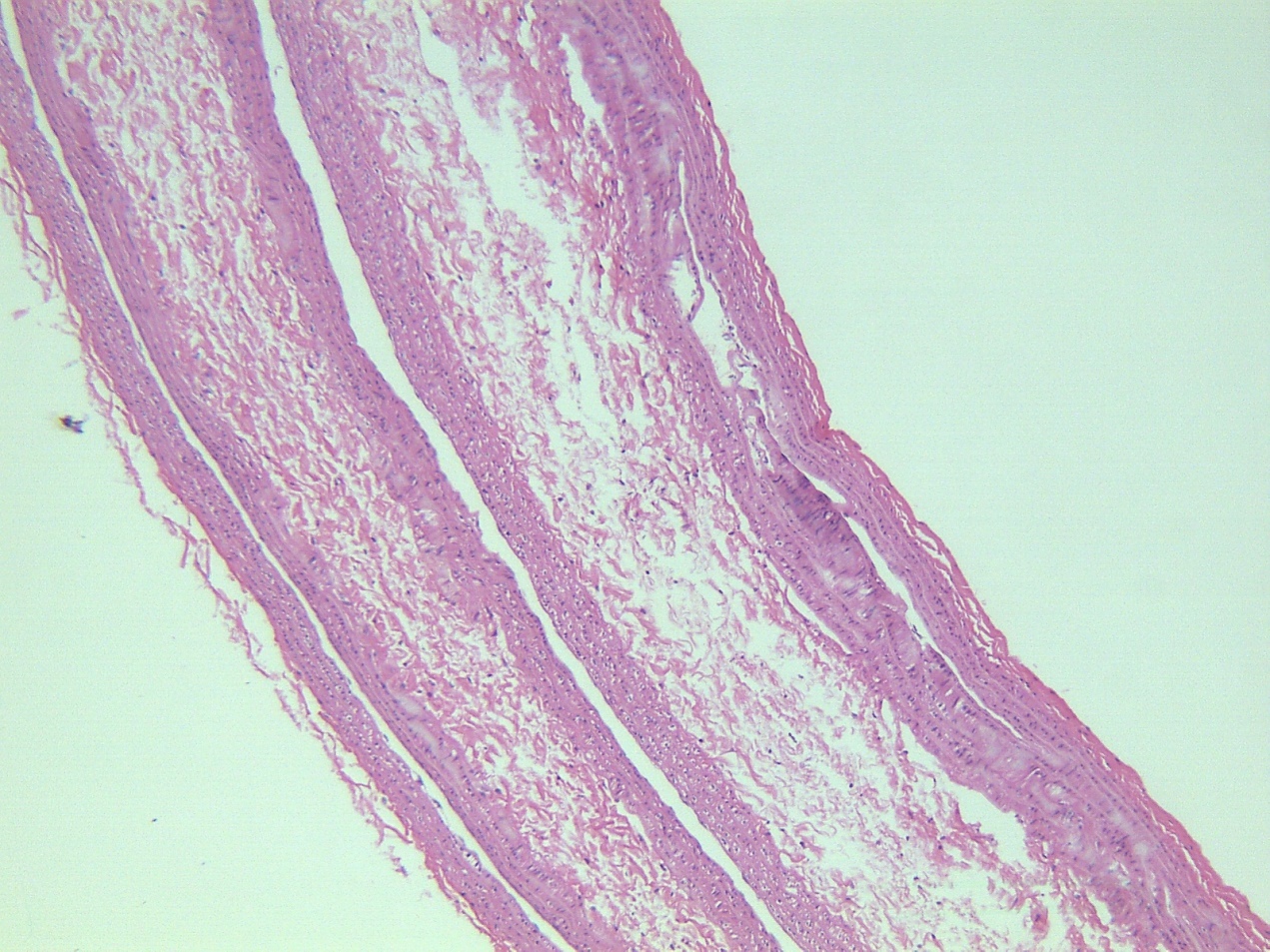


Figure1A, Left carotid artery (400×):


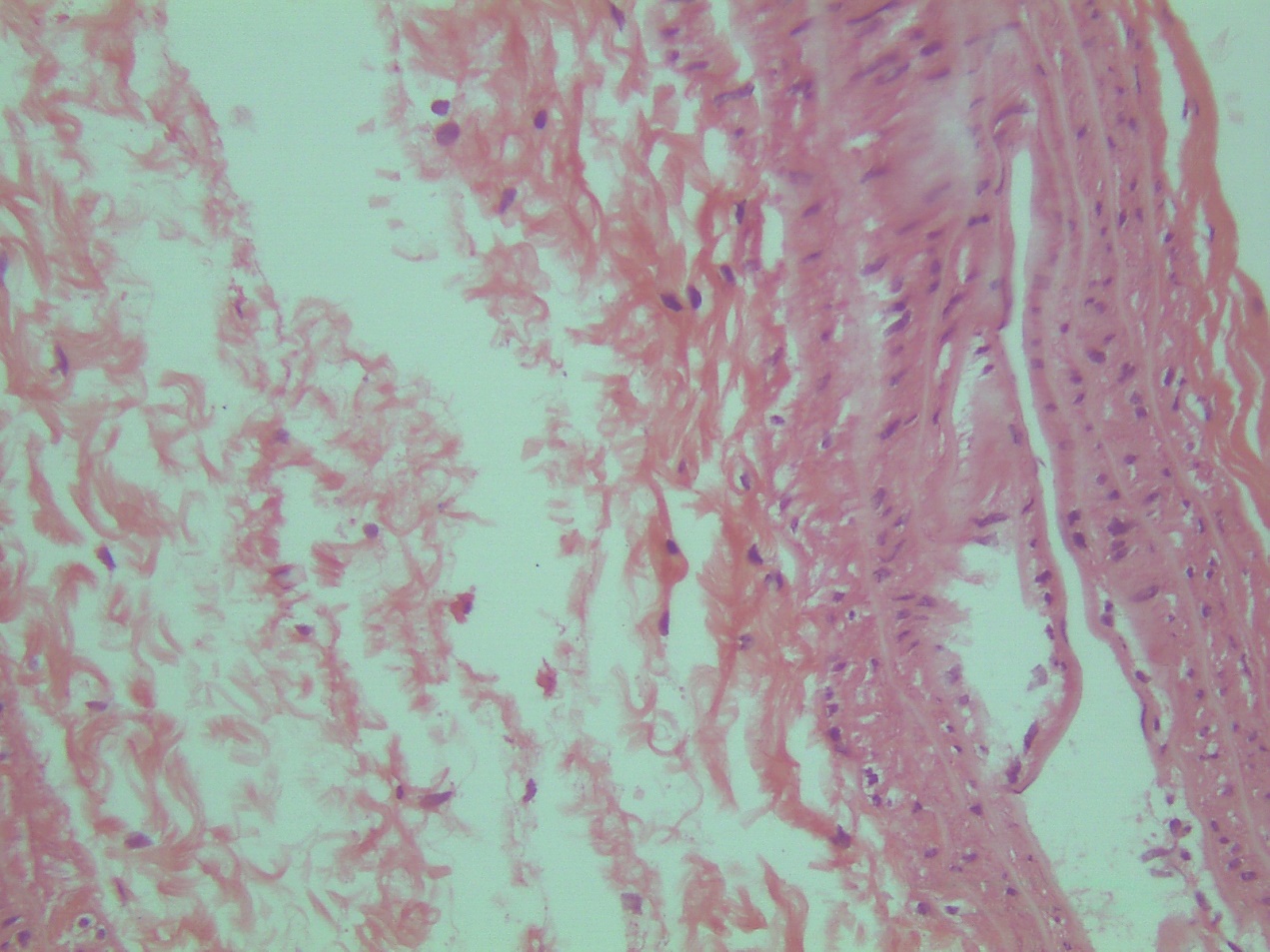


2. Figure2A&Figure3A, RT-PCR:


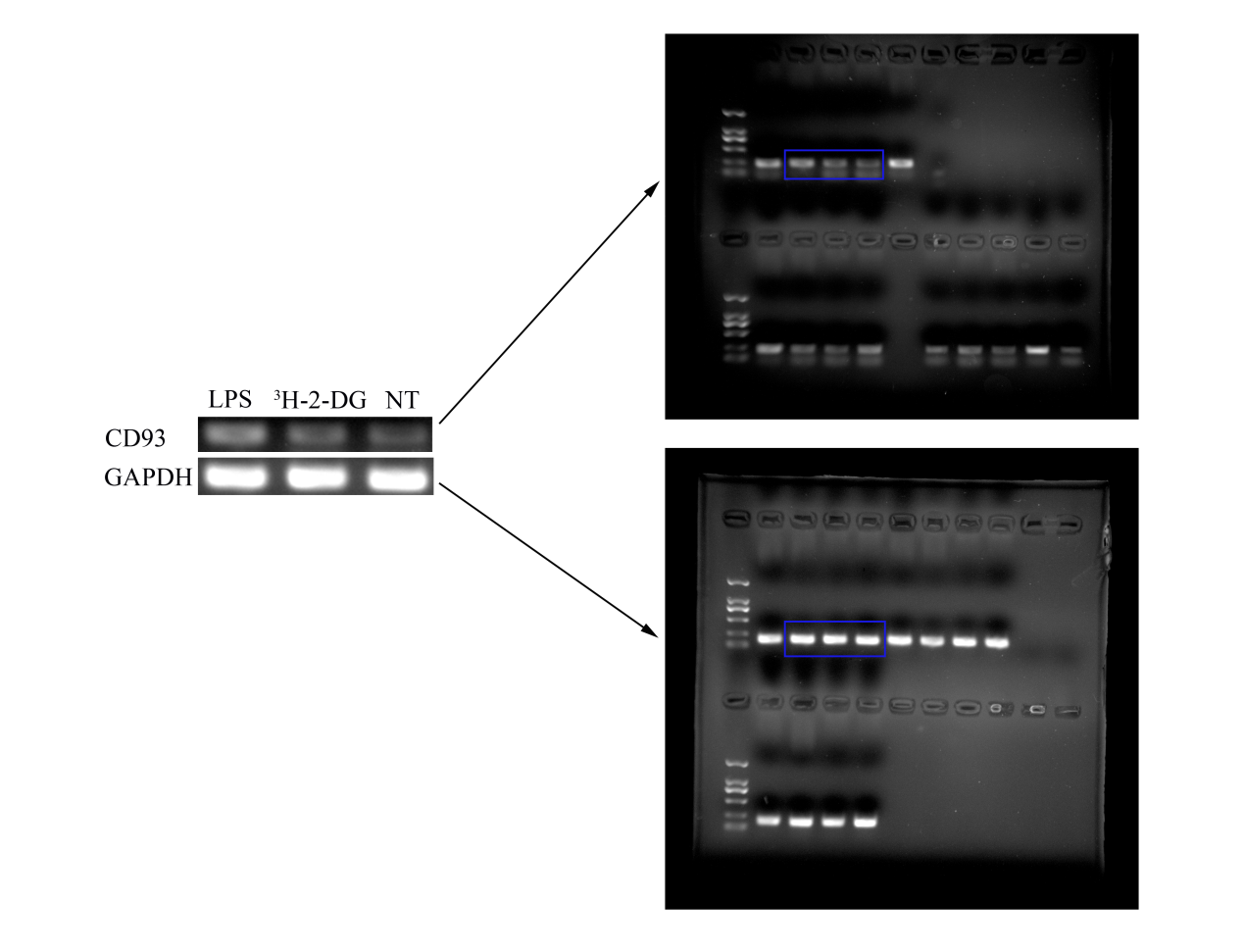


3. Figure2B&Figure3B, Western Blot:


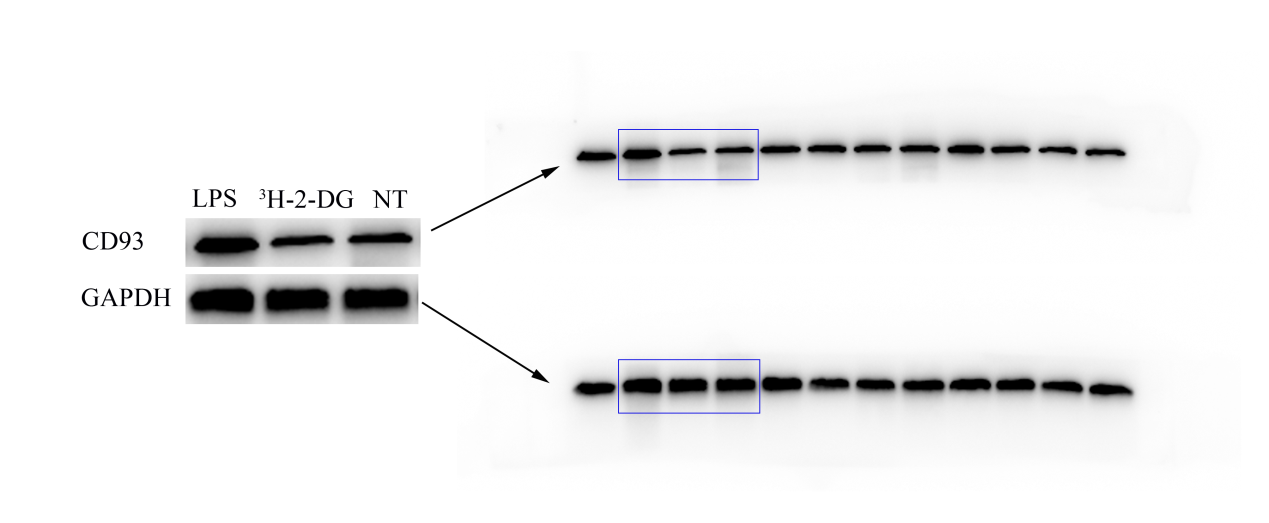


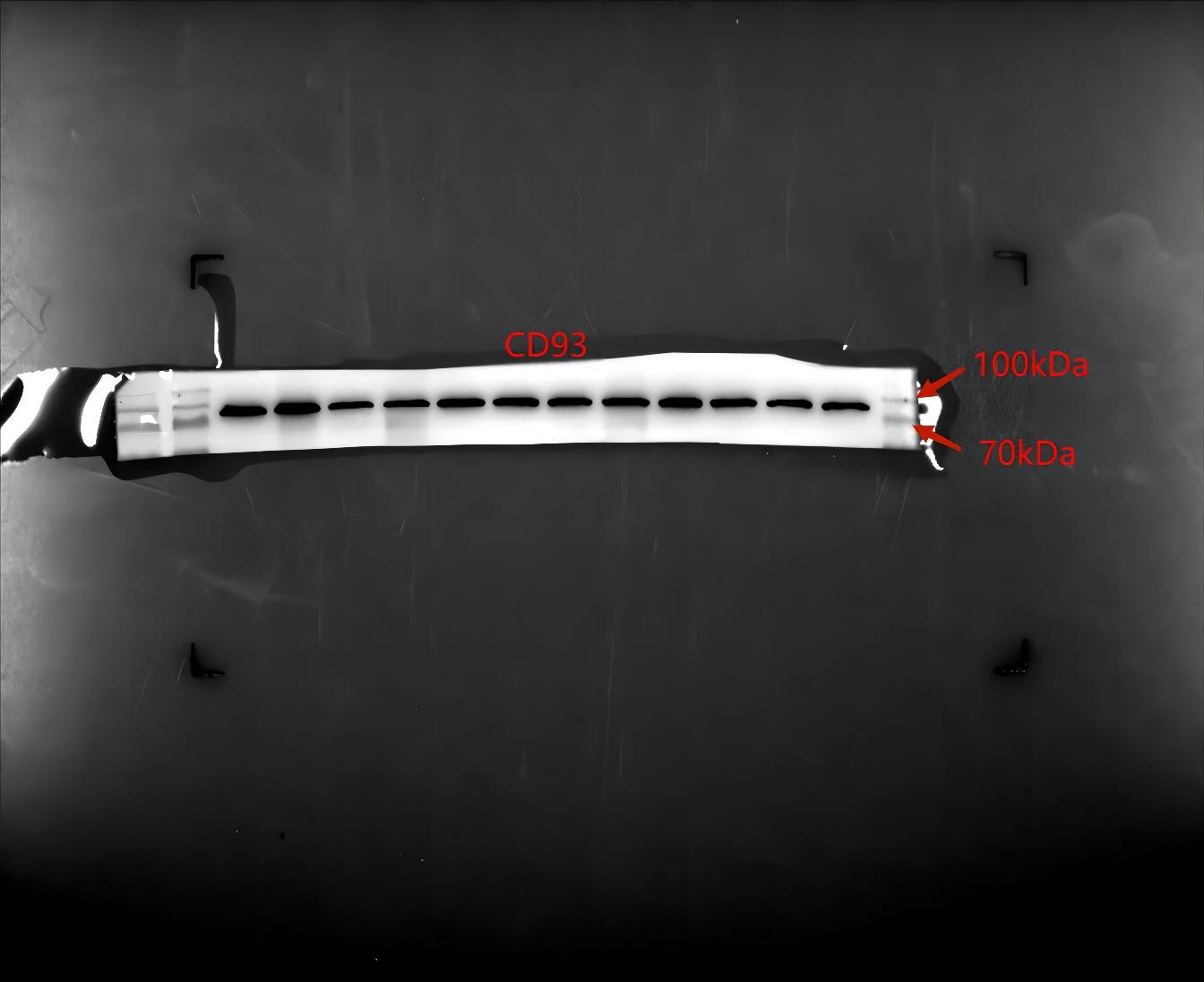


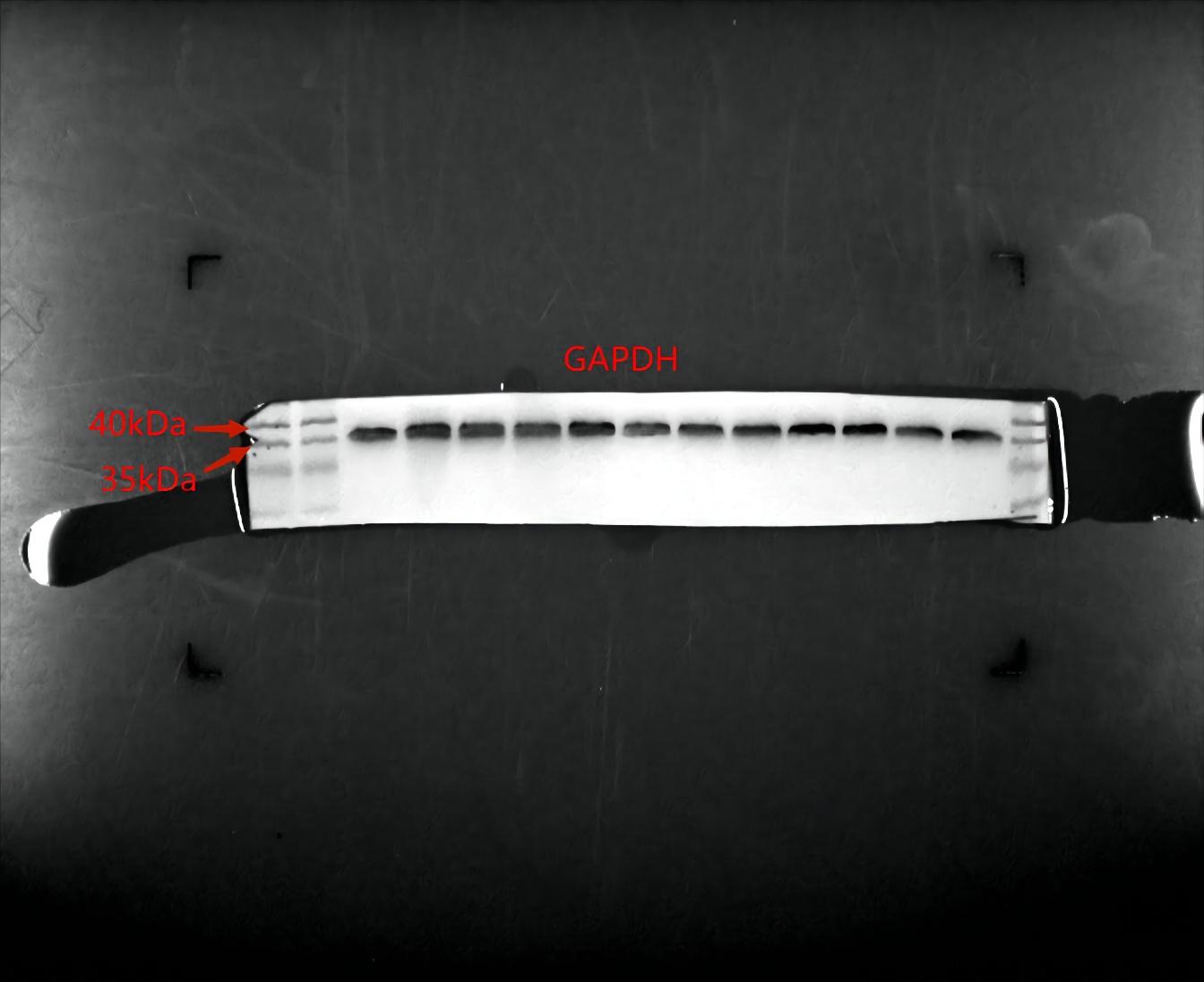


Note: The GAPDH strip was shot at a different exposure level when the marker was taken, so the color looks slightly lighter.

4. Figure2C&Figure3C, Phagocytosis of Dil-Ox-LDL:

NT:


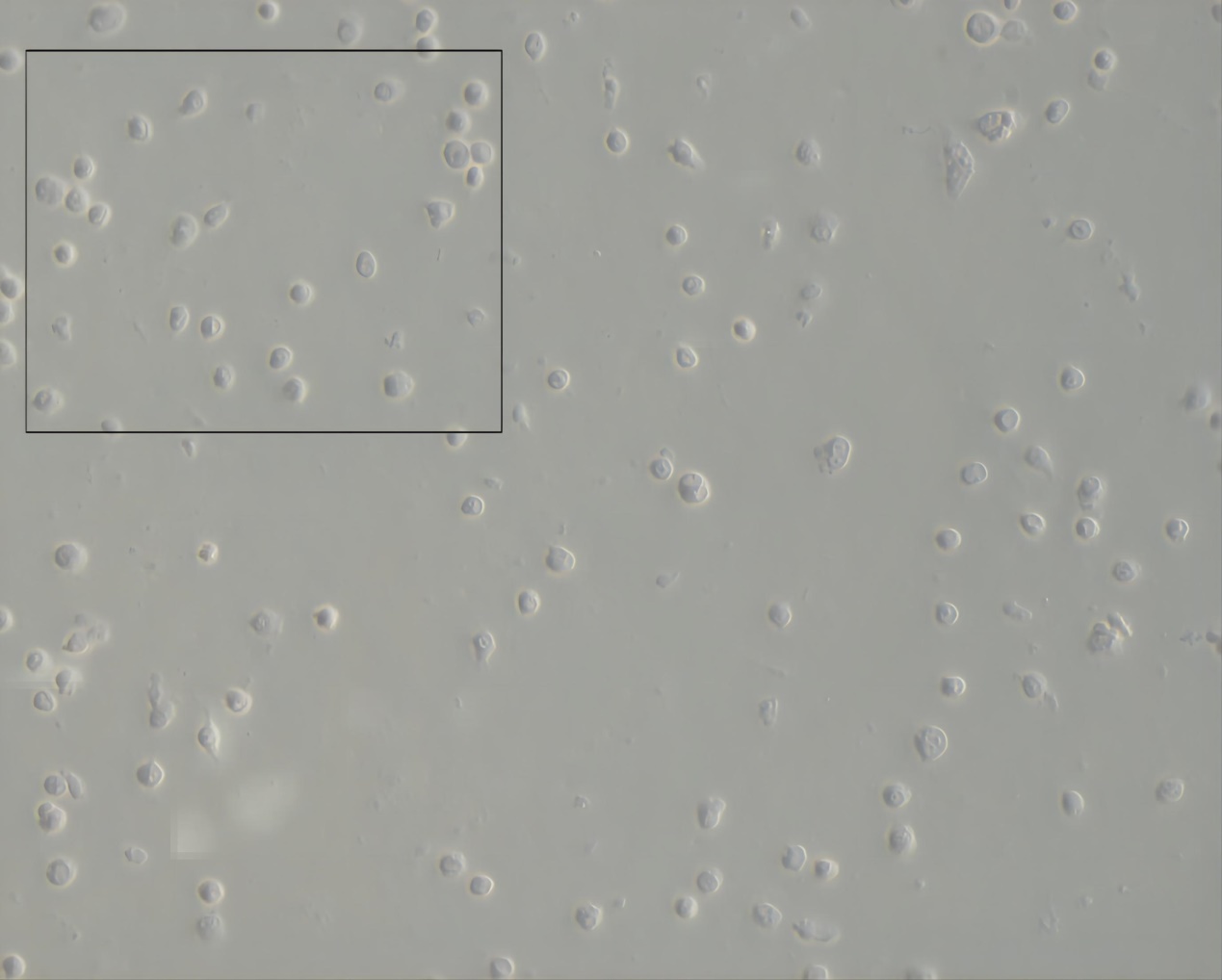


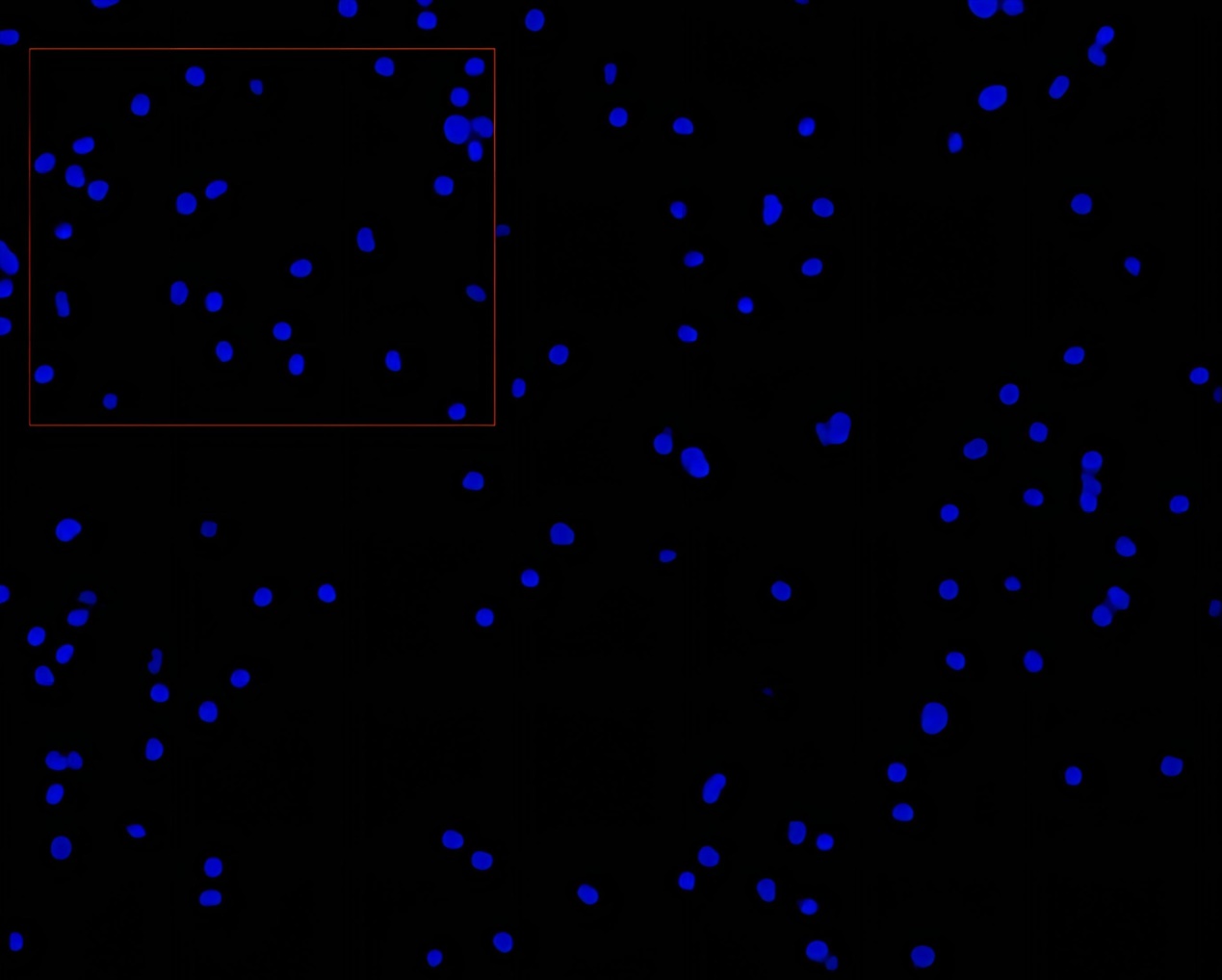


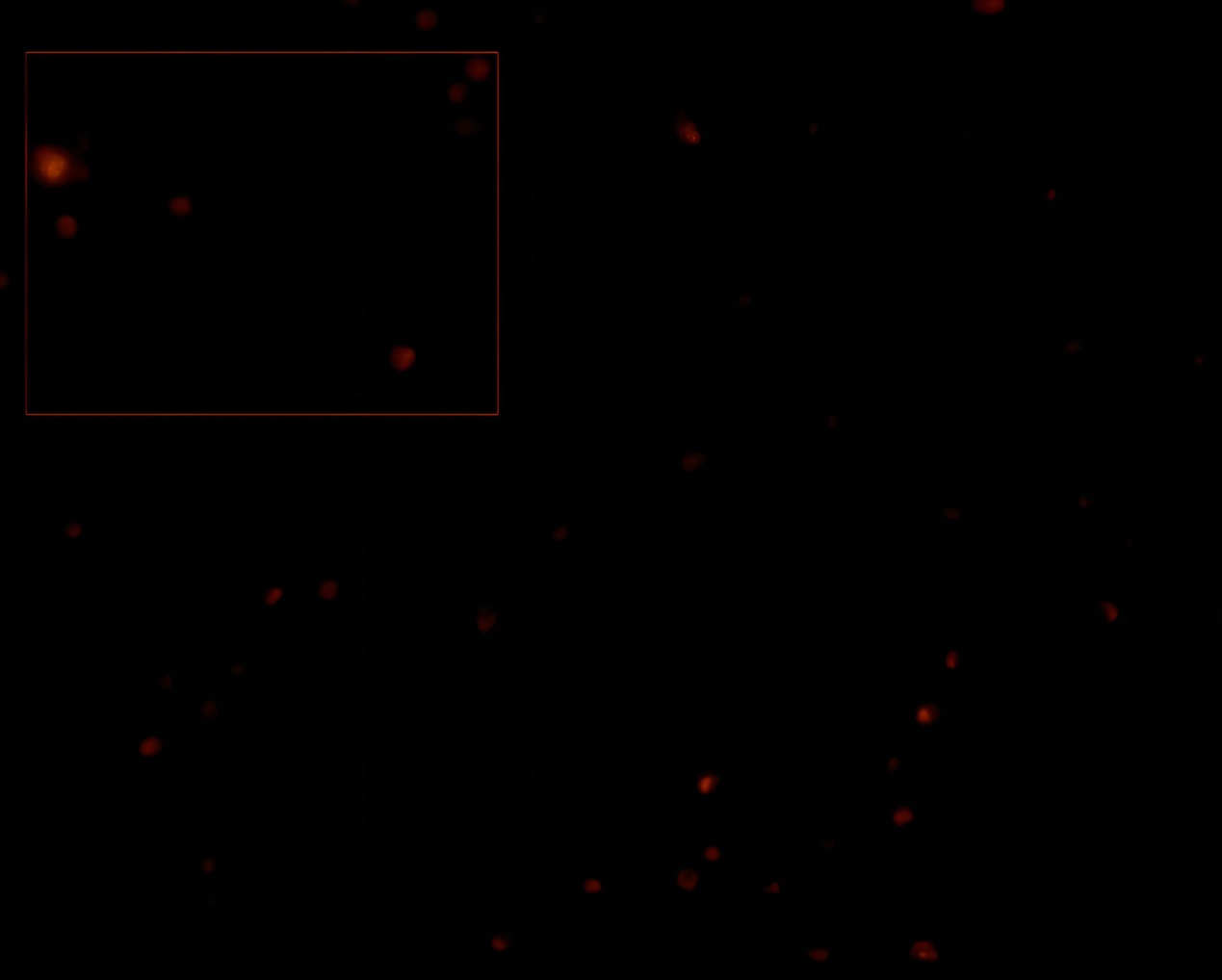


LPS:


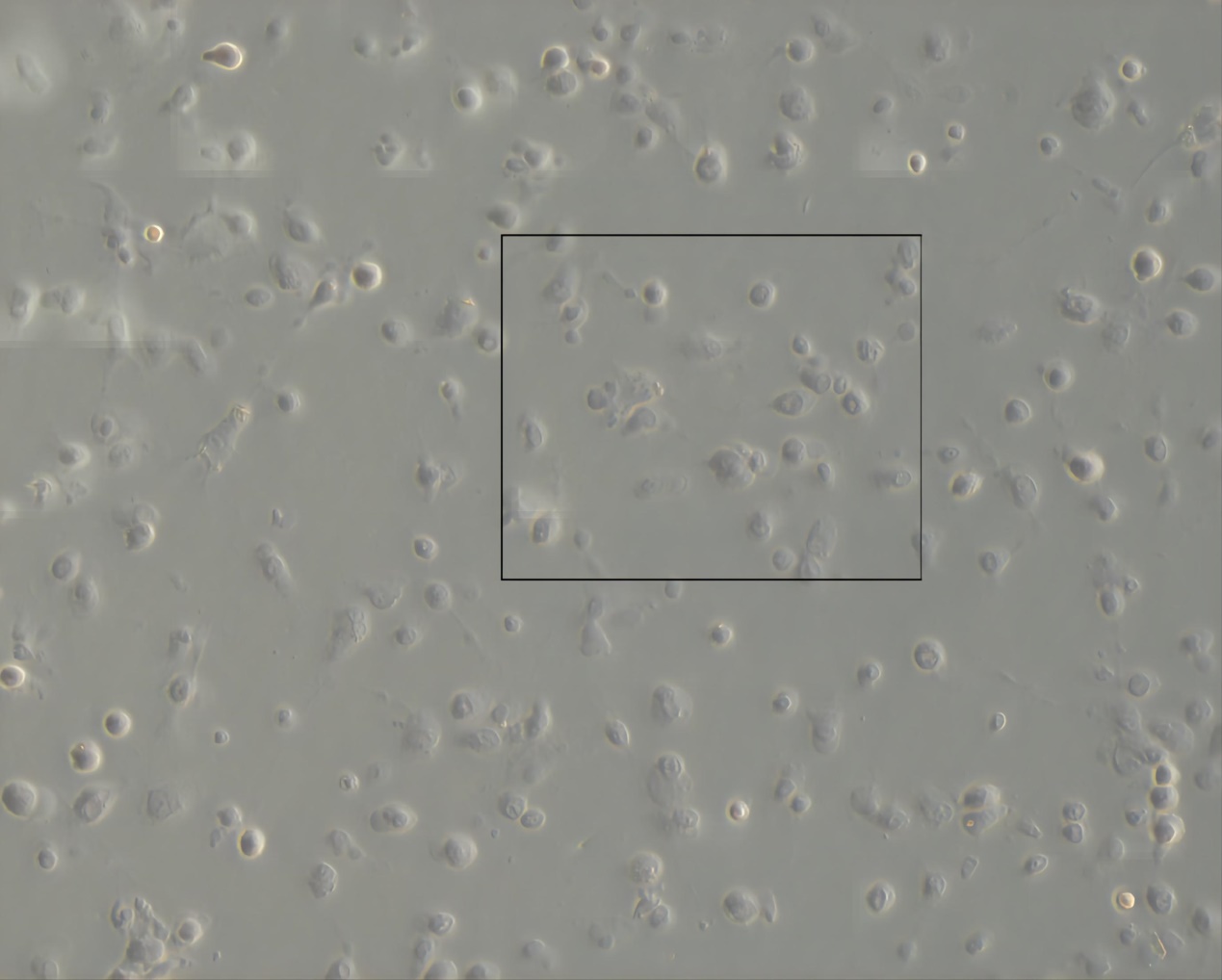


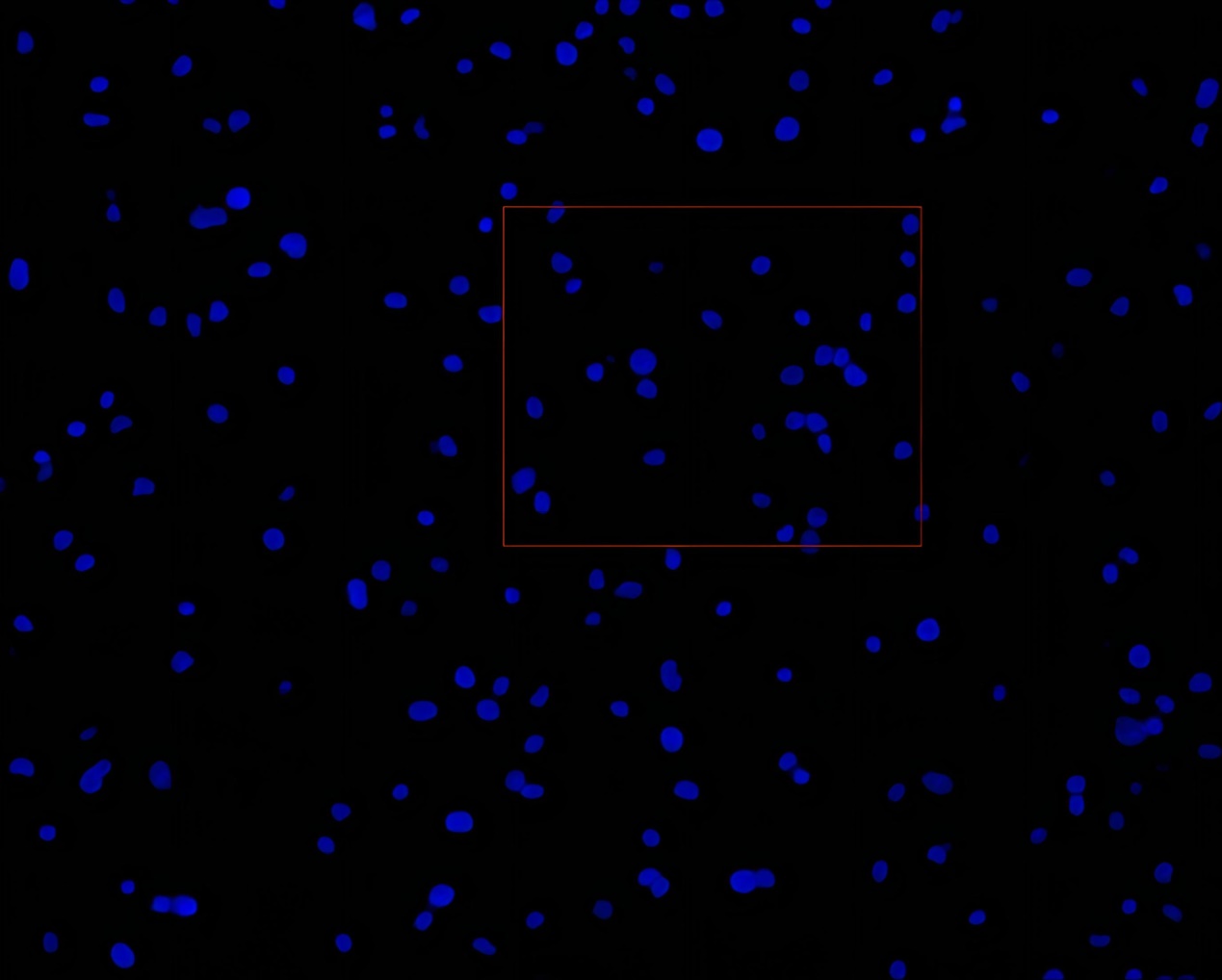


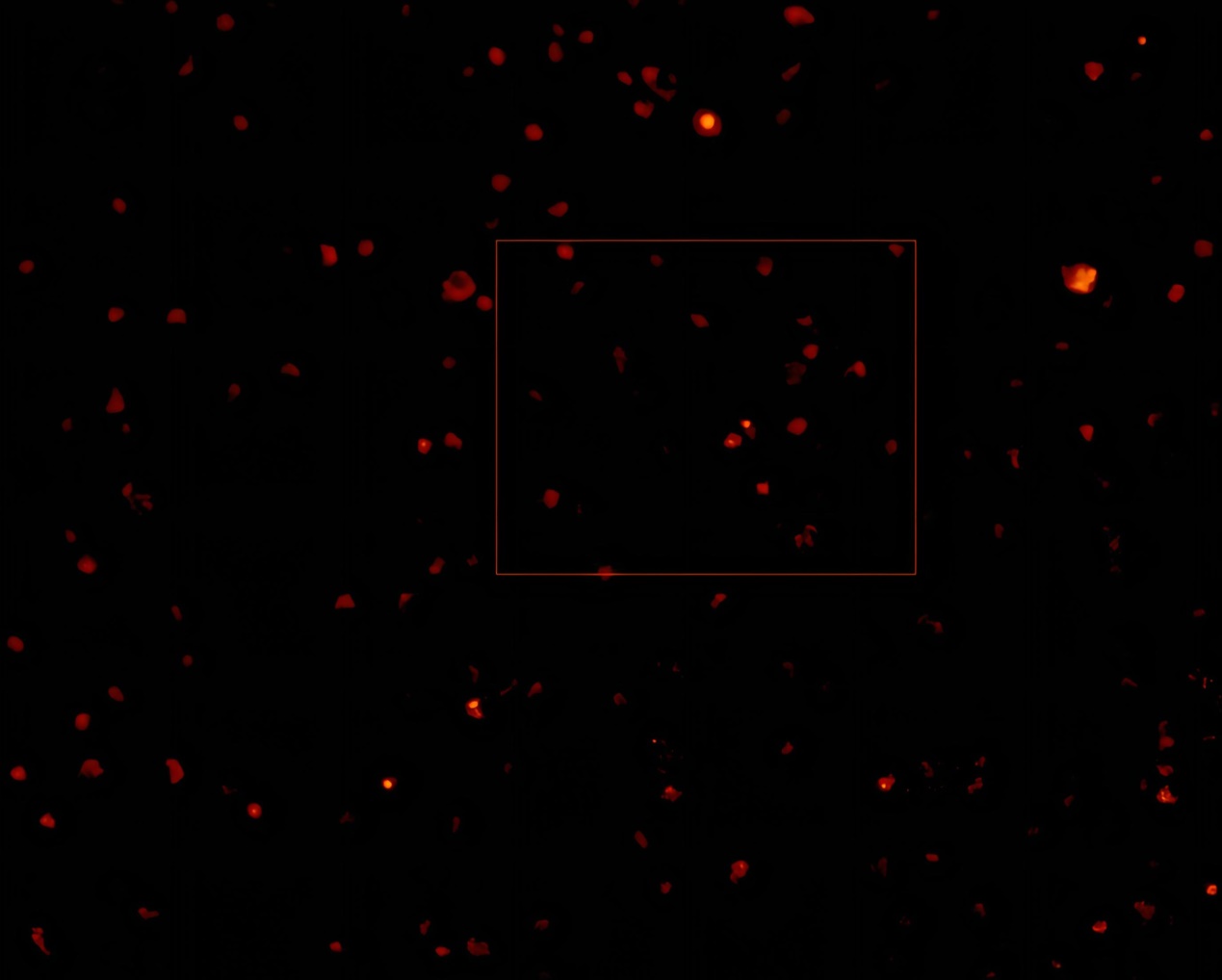


^3^H-2-DG:


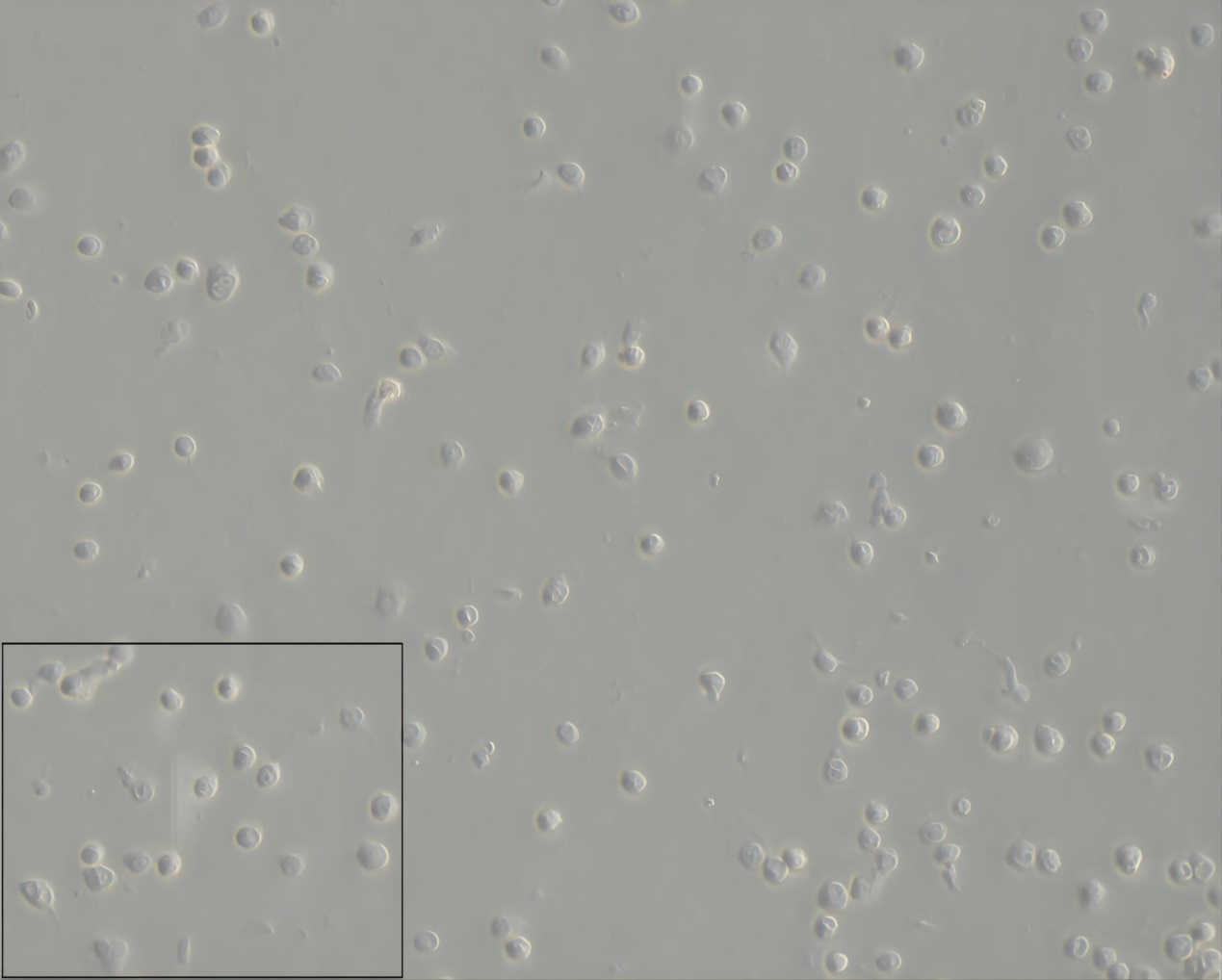


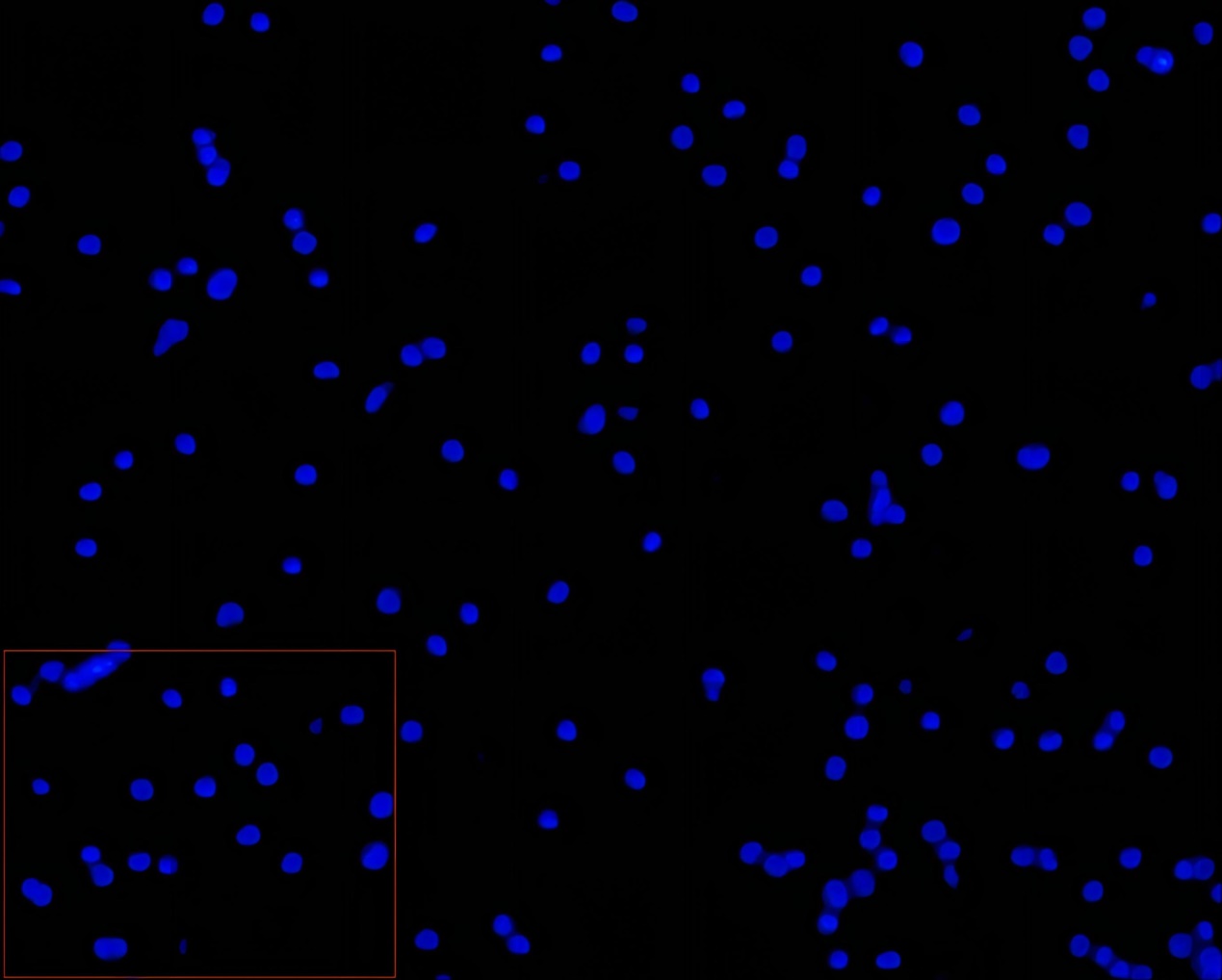


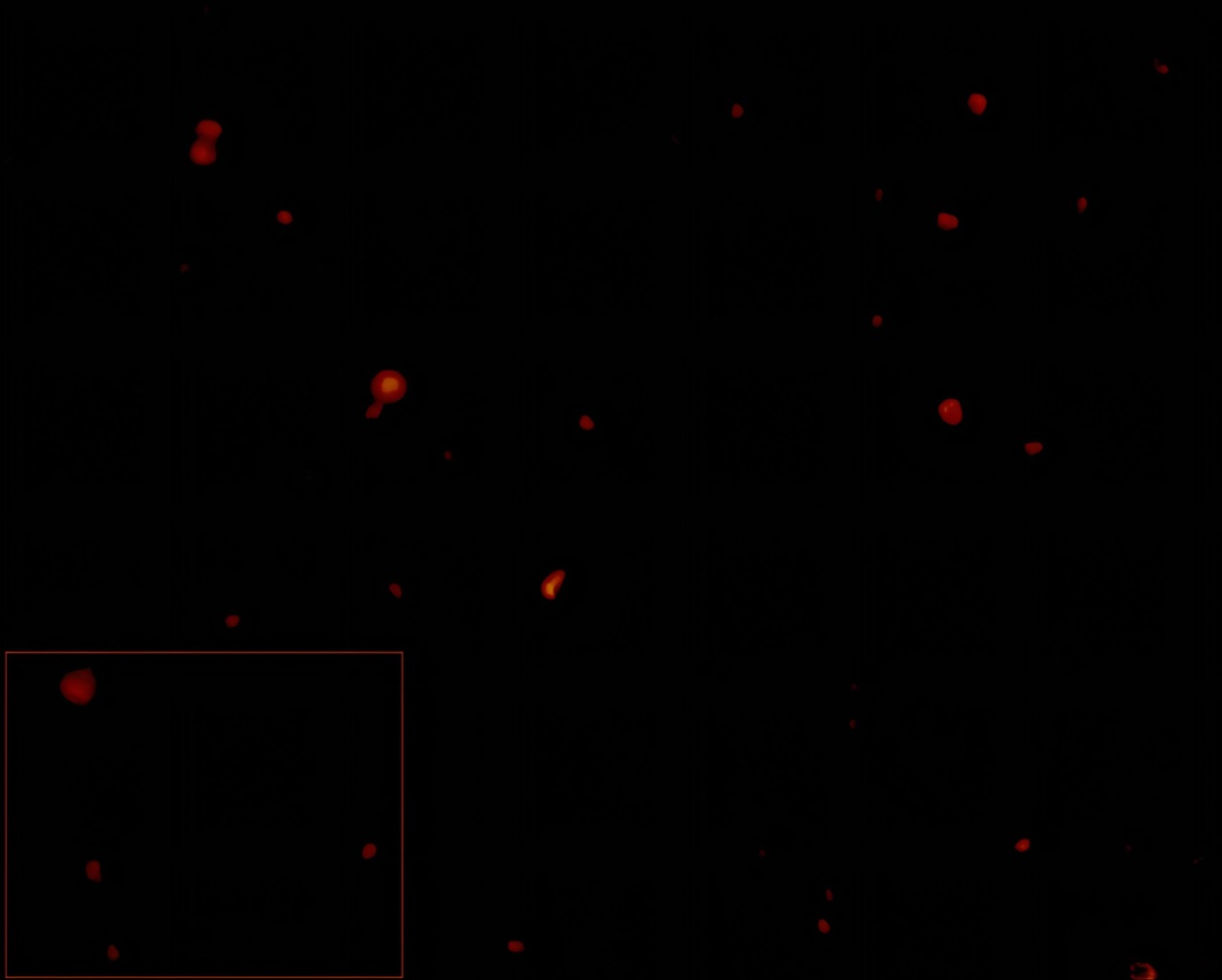


5. Figure2D&Figure3D, Morphology:

NT:


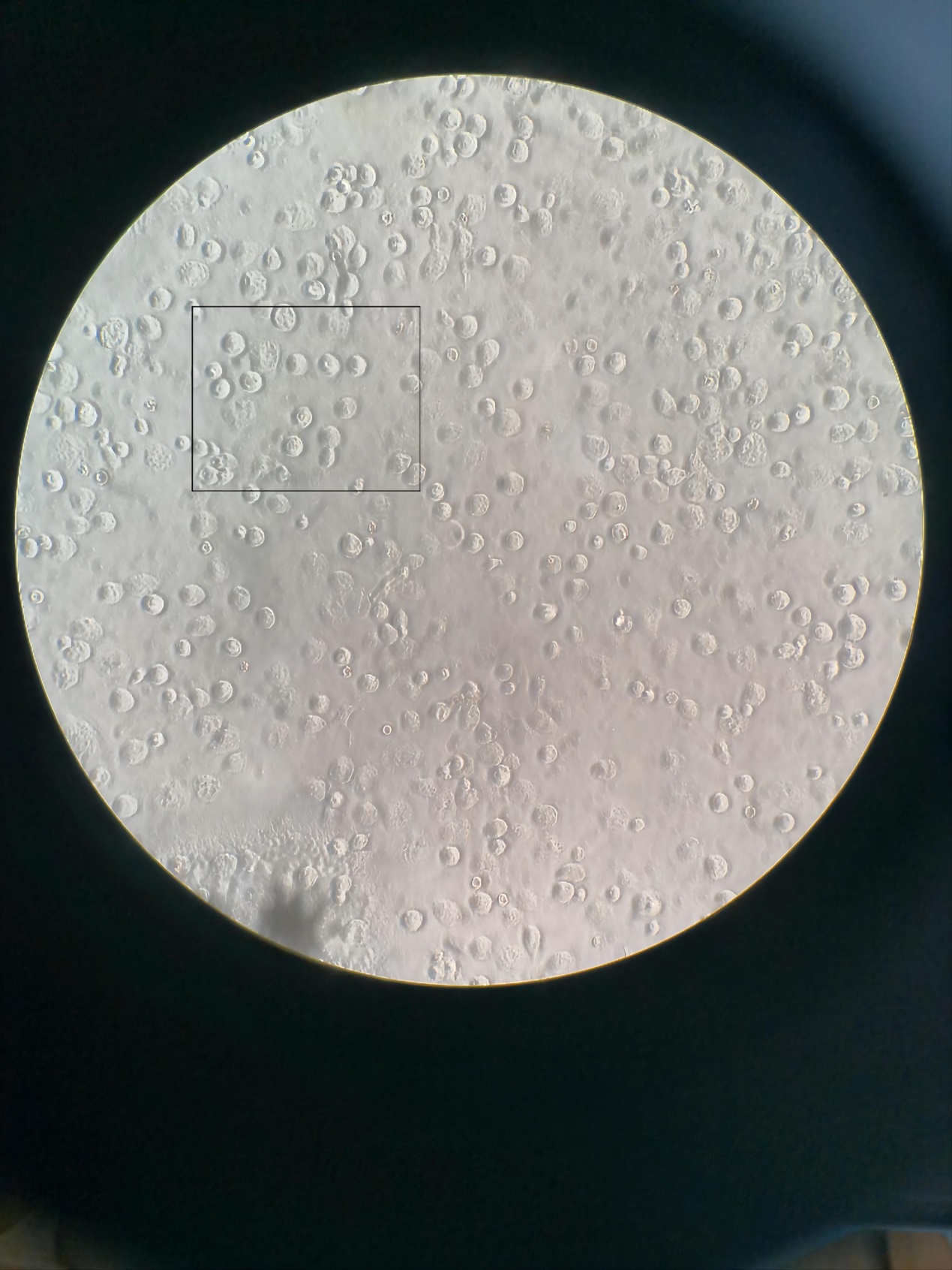


LPS:


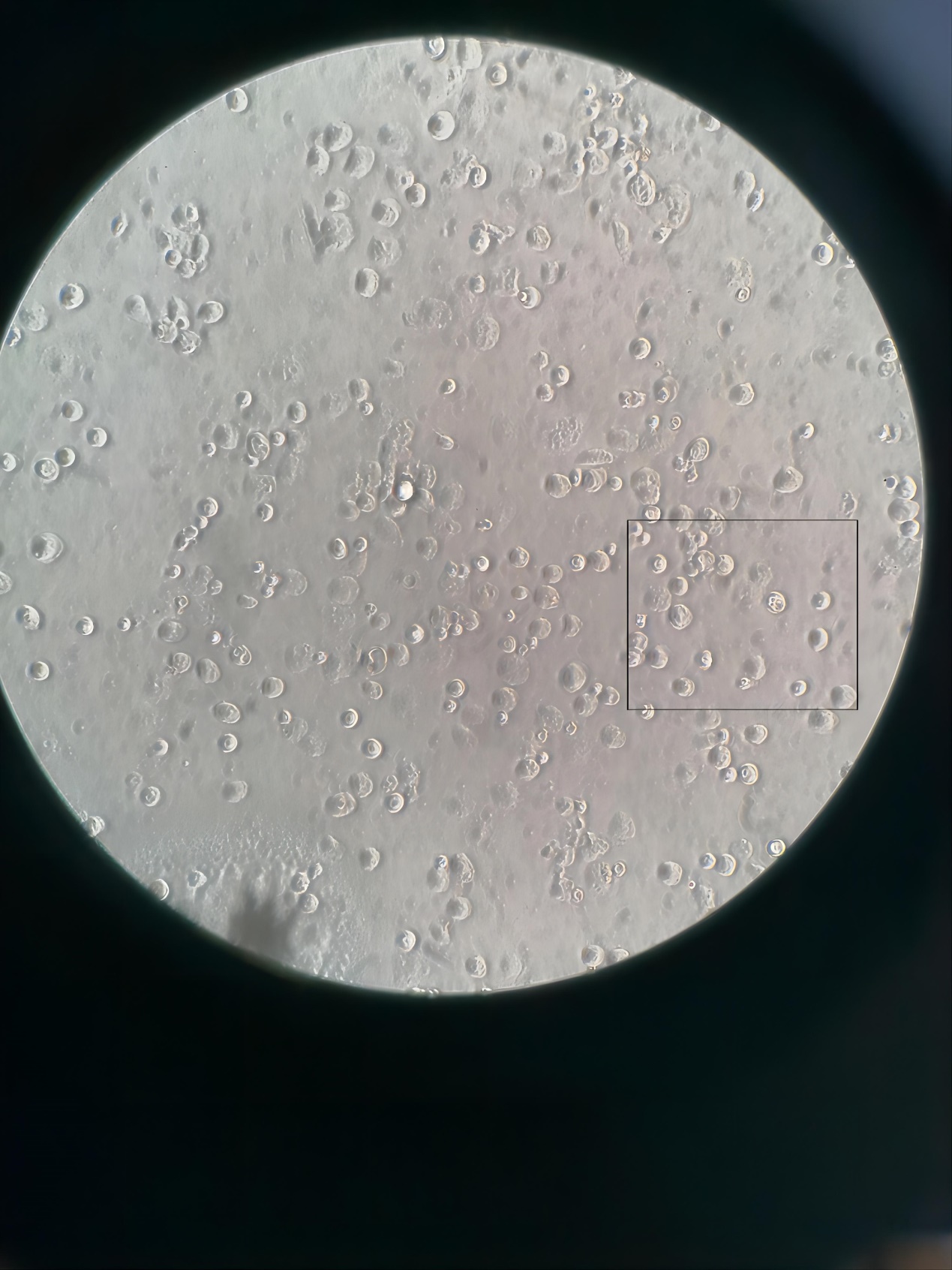


^3^H-2-DG:


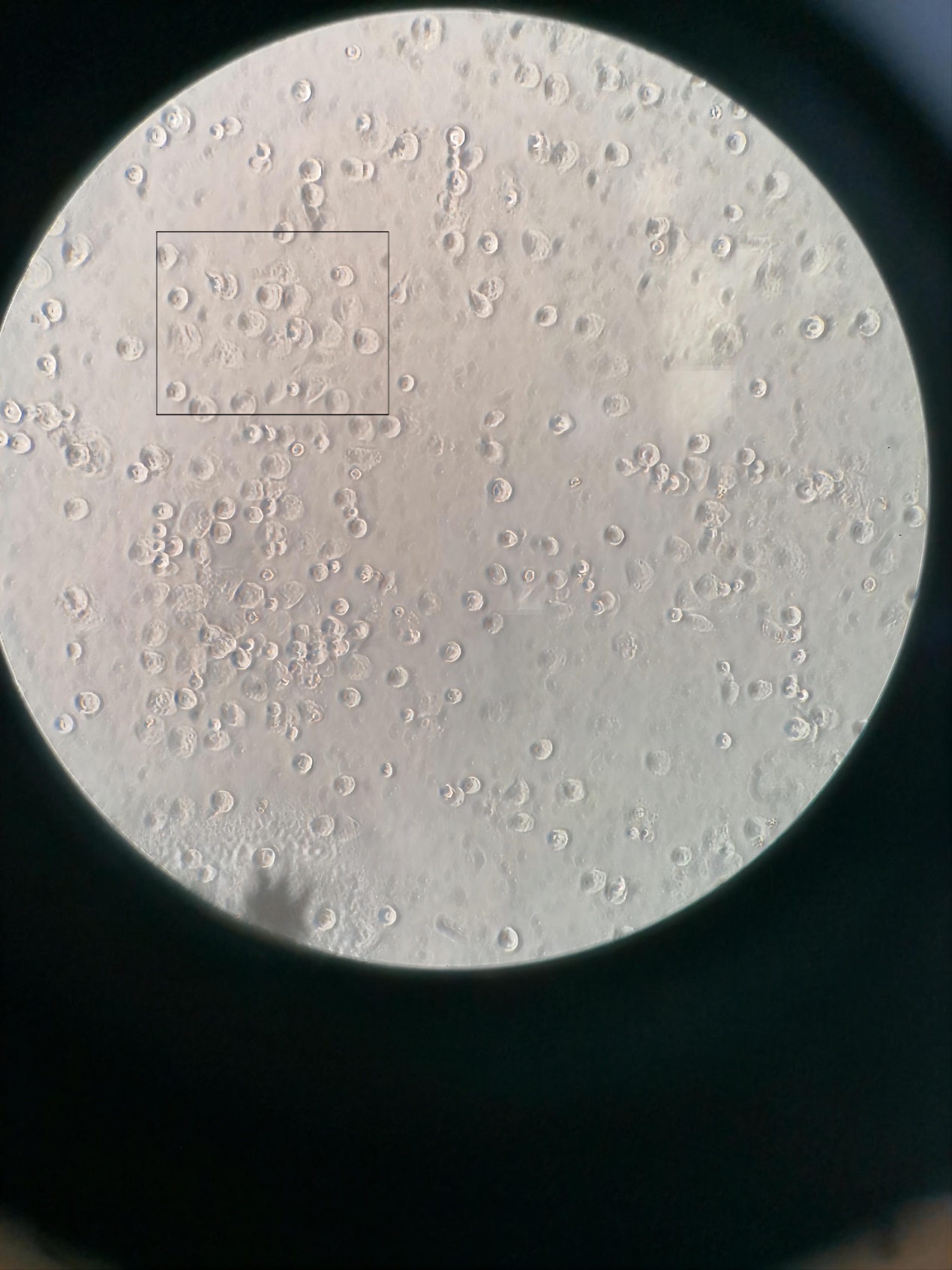


6. Figure5A, Tritium-phosphor-autoradiography of ex vivo aorta arch:

Autoradiography:


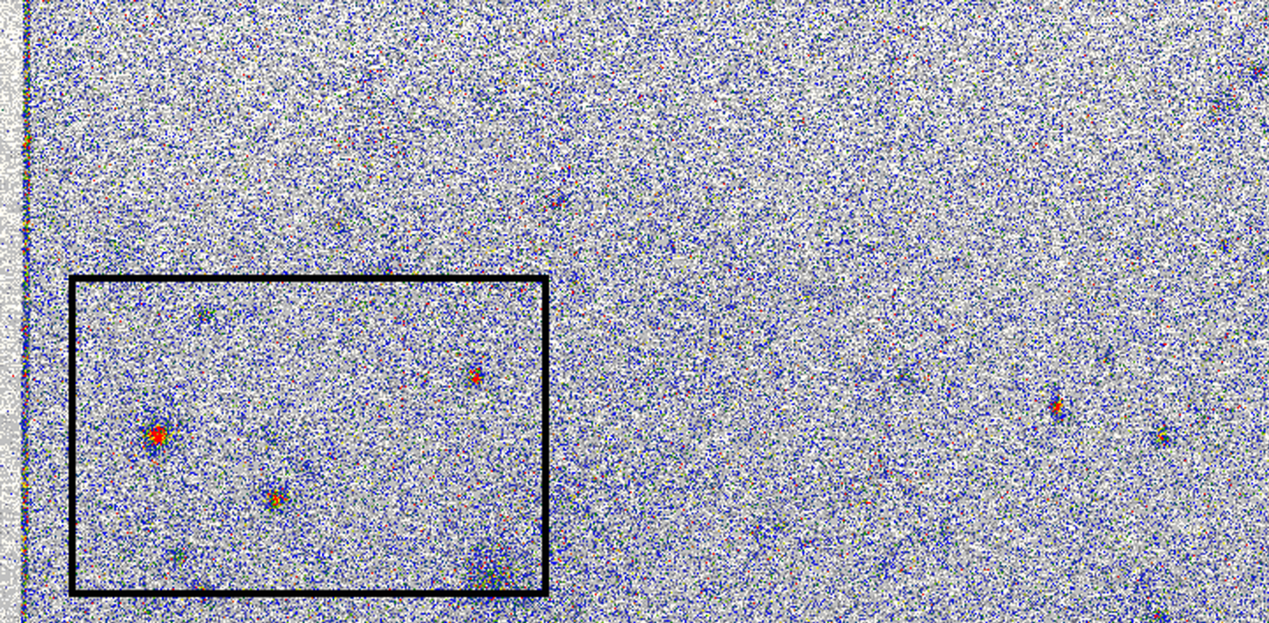


Aorta arch:


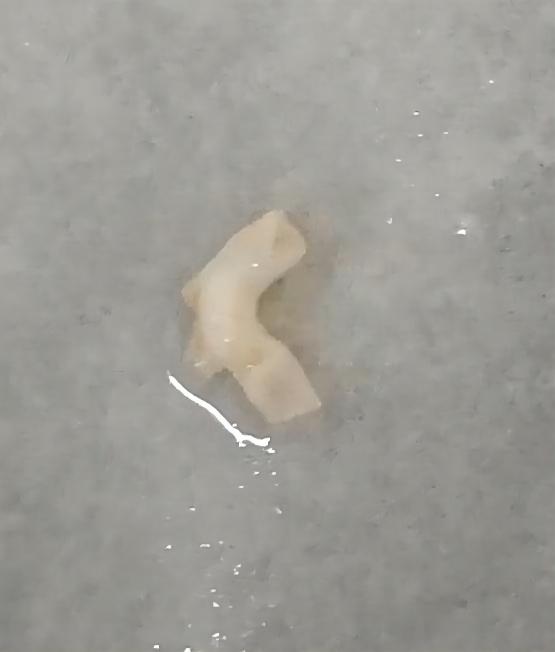


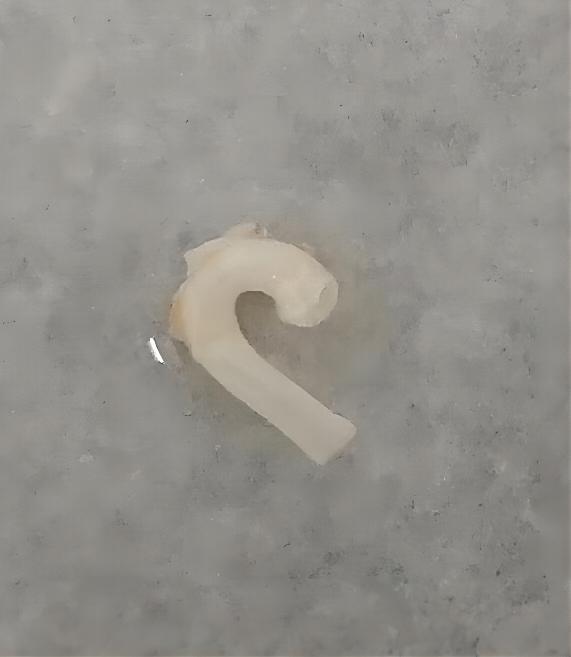


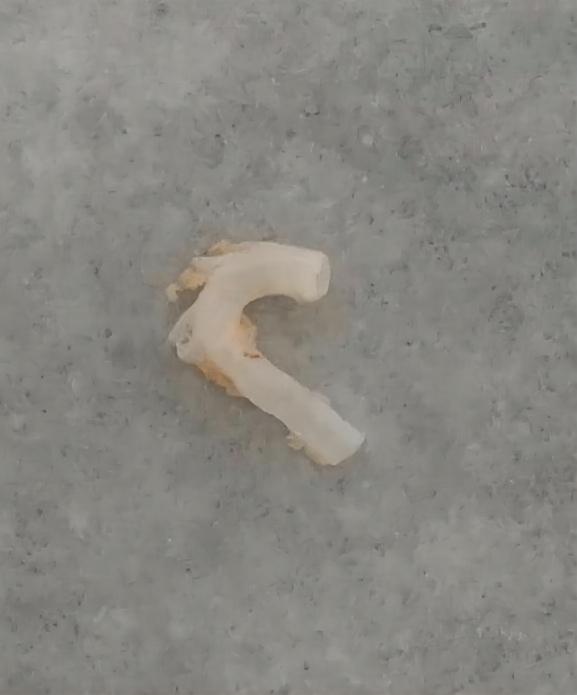

Supplement: Supplementary file 2 — Supplementary Material [file JCMM-26-2152-s001.docx]
